# RNA N^6^-methyladenosine demethylase FTO promotes diabetic wound healing through TRIB3-mediated autophagy in an m^6^A-YTHDF2-dependent manner

**DOI:** 10.1038/s41419-025-07494-3

**Published:** 2025-03-29

**Authors:** Zheng Dong, Shiyan Li, Yumeng Huang, Tianzhe Chen, Youjun Ding, Qian Tan

**Affiliations:** 1 Department of Burn and Plastic Surgery, Nanjing Drum Tower Hospital, Affiliated Hospital of Medical School, Nanjing University, Nanjing, Jiangsu 210008 China; 2 Department of Burns and Plastic Surgery, Nanjing Drum Tower Hospital Clinical College of Jiangsu University, Nanjing, Jiangsu 210008 China

**Keywords:** Molecular biology, Pathogenesis

## Abstract

N^6^-methyladenosine (m^6^A) RNA modification impaired autophagy results in delayed diabetic wound healing. In this study, it was found that fat mass and obesity-associated protein (FTO) was significantly downregulated in the epidermis of diabetic patients, STZ-induced mice and db/db mice (type I and II diabetic mice) with prolonged hyperglycemia, as well as in different types of keratinocyte cell lines treated with short-term high glucose medium. The knockout of FTO affected the biological functions of keratinocytes, including enhanced apoptosis, inhibited autophagy, and delayed wound healing, producing consistent results with high-glucose medium treatment. High-throughput analysis revealed that tribbles pseudokinase 3 (TRIB3) served as the downstream target gene of FTO. In addition, both in vitro and in vivo experiments, TRIB3 overexpression partially rescued biological functions caused by FTO-depletion, promoting keratinocyte migration and proliferation via autophagy. Epigenetically, FTO modulated m^6^A modification in the 3’UTR of TRIB3 mRNA and enhanced TRIB3 stability in a YTHDF2-dependent manner. Collectively, this study identifies FTO as an accelerator of diabetic wound healing and modulates autophagy via regulating TRIB3 in keratinocytes, thereby benefiting the development of a m^6^A-targeted therapy for refractory diabetic wounds.

## Introduction

Impaired wound healing is a common and serious complication of diabetes. Moreover, it is associated with increased morbidity and mortality, as well as adverse impacts on a patient’s functional ability and life quality [1]. Diabetic foot ulcers (DFU) normally result in a chronic wound due to the impairment of healing, which may lead to permanent loss of function, amputation, and even death [2, 3]. Many factors lead to the poor wound healing ability of diabetes, including sensory neuropathy, vascular insufficiency, hypoxia, chronic inflammation, and wound infection. Most of these factors are caused by hyperglycemic conditions [4]. The epidermis, the outermost layer of the skin, makes a critical difference in wound closure. During the wound healing process, epidermal cells migrate to cover the wound and proliferate to reform the epidermal barrier [5–8]. However, it has been shown that the thickness of diabetic epidermis layer was significantly thinner than control [9, 10]. Epidermis thinning may be a key factor in delayed diabetic wound healing. Keratinocytes are the major cell type in the epidermis. In recent years, it has been found that the physiologic impairment of epidermal keratinocytes plays a crucial role in the poor healing ability of diabetic wounds [11]. Factors involving keratinocytes that may contribute to abnormal damage repair and wound healing in diabetes include migratory and proliferative dysfunction.

Several studies have shown that genetic and epigenetic regulation can influence cell growth and division. N^6^-methyladenosine (m^6^A) is the most abundant and universally found RNA modification in transcripts, which affects the production and metabolism of RNA, such as mRNA processing and decay, translation, transcription, and RNA-protein interactions [12, 13]. M^6^A RNA modifications are proven to play critical roles in controlling skin biology and disease pathogenesis, and m^6^A eraser fat mass and obesity-associated protein (FTO) also plays a key role in regulating skin-related diseases such as melanoma [14], scleroderma [15], and psoriasis [16]. Emerging evidence suggests that FTO affects cell survival and death by inducing apoptosis and autophagy [14, 17, 18]. Autophagy is an important biological process that plays a crucial role in maintaining cellular balance and physiology. The aberrant regulation of autophagy is involved in the pathogenesis of skin diseases, ranging from wound healing to dermatitis to skin cancer. Recently, research has demonstrated that high-glucose suppresses autophagy in keratinocytes, resulting in non-healing or slow-healing wounds in diabetes [19, 20]. Furthermore, in vivo studies revealed that autophagy impairment inhibited wound closure. However, the mechanism through which autophagy dysregulation leads to wound healing remains unclear. In autophagy, SQSTM1 (sequestosome 1) is a crucial protein marker that regulates autophagy selection. In general, the expression of SQSTM1 is regulated by autophagy. In turn, SQSTM1 is also involved in the regulation of autophagy. Evidence has already shown that SQSTM1 contributes to the dysregulation of autophagy in diabetic wound healing [21–23]. A comprehensive understanding of the molecular mechanism of autophagy-modulating in keratinocytes was critical to finding potential therapeutic targets for diabetic wound healing. Nonetheless, the potential involvement of FTO via autophagy in diabetic skin still needs further investigation.

Herein, we identified FTO-mediated m^6^A as a critical regulator of wound healing in diabetic wounds (DW). We discovered that FTO is specifically downregulated in diabetic epidermis compared to other m^6^A regulators. Further functional experiments showed that FTO was essential for keratinocyte proliferation and migration through autophagic regulation. Using RNA-sequence (RNA-seq) and functional analysis, we demonstrated tribbles pseudokinase 3 (TRIB3) as a potential downstream target of FTO-mediated m^6^A modification. Overexpression of TRIB3 significantly rescued cell proliferation and migration in vitro with high-glucose medium treatment and promoted wound healing in vivo with hyperglycemic conditions. Furthermore, we demonstrated that YTHDF2 recognized and bounded to the m^6^A site in TRIB3 mRNA and modulated its degradation. Together these investigations uncovered a novel epigenetic regulatory mechanism of diabetic wound healing by which the m^6^A eraser FTO facilitated keratinocyte proliferation and migration by elevating TRIB3 mRNA stability in a YTHDF2-dependent manner.

## Results

### FTO is downregulated, while m6A is upregulated in diabetic epidermal keratinocytes

**Fig. 1:**
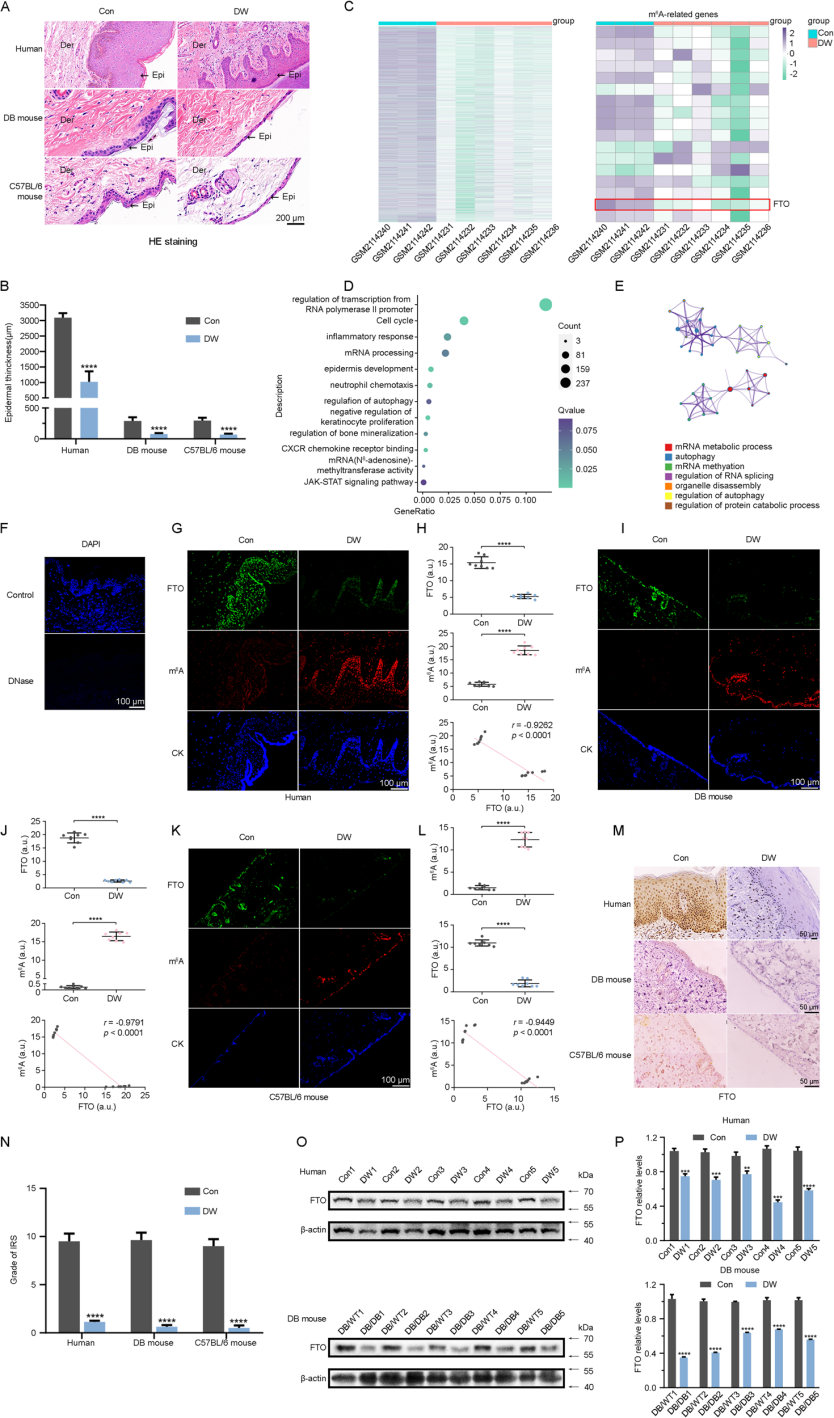
FTO is downregulated, while m^6^A is upregulated in the diabetic epidermis. **A** and **B** Representative HE staining of the diabetic wound (DW) and the control (Con) in samples of humans, DB-strain mice, and C57BL/6 mice. Quantification of epidermal thicknesses (*n* = 5 per group, Scale bar, 200 μm). **C**Heatmaps showed differentially expressed genes (DEGs) and m^6^A-related DEGs in DW and Con, respectively. **D** Functional enrichment analysis of DEGs. **E** The PPI networks were constructed by the STRING database for modules with a threshold value > 0.4. **F**Nuclei are stained with DAPI in blue in normal human skin treated with or without DNase I (Scale bar, 100 μm). **G, I, K** Immunofluorescence staining of FTO (green), m^6^A (red), and keratin (blue) in Con (*n* = 5) and DW (*n* = 5). G, Human tissue, I, DB-strain mice tissue (Con, DB/WT mice; DW, DB/DB mice), and K, C57BL/6 mice tissue (Scale bar, 100 μm). **H, J, L** Quantification of FTO and m^6^A levels using Image J and the negative correlation of m^6^A levels with FTO levels by Spearman’s test. **M, N** IHC analysis of FTO in the epidermis of DW and the control group in humans, DB-strain mice (Con, DB/WT mice; DW, DB/DB mice), and C57BL/6 mice (Scale bar, 50 μm). Quantitative analysis of FTO protein (brown color) levels in the epidermis was performed according to the IRS (*n* = 5). **O, P**Immunoblot analysis of FTO between DW and Con in humans and DB-strain mice. The quantification results of FTO protein expression are shown. The data are presented as the mean ± SD or mean ± SEM (**B, H, J, L, N, P**), and *P* values of all data by a two-tailed unpaired t-test are indicated. *P < 0.05, **P < 0.01, ***P < 0.001, ****P < 0.0001.

To identify whether m^6^A RNA methylation and its regulators are responsible for the thinner epidermis in diabetics. In addition to the human skin samples, skin tissues from DB-strain mice and C57BL/6 mice were also collected and analyzed. The diabetic epidermis was obviously thinner than normal in both humans and different strains of mice (Fig. 1A, B). To explore the gene expression profiles of the m^6^A-related regulators in diabetic tissue, we performed data mining from GSE80178 and found that there were significant differences in m^6^A-related genes between diabetic epidermis and normal. Indeed, the expression of FTO mRNA was significantly down-regulated in diabetic skin (Fig. 1C). Functional enrichment analysis indicated that differentially expressed genes (DEGs) in GSE80178 were significantly enriched in m^6^A methylation, autophagy, and the inflammatory response (Fig. 1D, E). To detect the expression pattern of m^6^A more accurately, all samples were treated with DNase I to degrade potential DNA (Fig. 1F). The diabetes epidermis showed downregulation of FTO and upregulation of m^6^A compared to the normal epidermis layer in humans (Fig. 1G, H). This phenomenon could also be found in type I and II diabetic mice (Fig. 1I–L). To further verify our findings, we performed immunohistochemistry (IHC) and western blot (WB) and obtained the same results, and the expression of FTO was significantly decreased in the diabetic epidermis (Fig. 1M–P).

To mimic the environment of diabetic epidermal cells in vitro, we exposed human keratinocyte cell lines Human Immortalized Epidermal Cells (HaCaT) and Normal Human Epithelial Keratinocytes (NHEK) to normal glucose (NG) and different glucose concentrations, while using mannitol as an osmotic control. We found that high levels of glucose decreased HaCaT and NHEK cell proliferation, but hyperosmotic stress had no appreciable effect (SFig. 1A-C). Meanwhile, HaCaT and NHEK cells with high-glucose treatment resulted in increased apoptosis rates compared with NG and the osmotic control (SFig. 1D, E). Moreover, cell migration capacity was reduced in HaCaT and NHEK cells with high-glucose treatment compared with NG and the mannitol control (SFig. 1F, G). This result suggested the impairment of keratinocyte migration in high-glucose. Indeed, the mRNA and protein expression levels of FTO were reduced in HaCaT and NHEK cells with high-glucose treatment but had no detectable effect in the normal-glucose group or the mannitol control (SFig. 1H-J).

### FTO-silenced inhibit proliferation and migration of keratinocytes

**Fig. 2:**
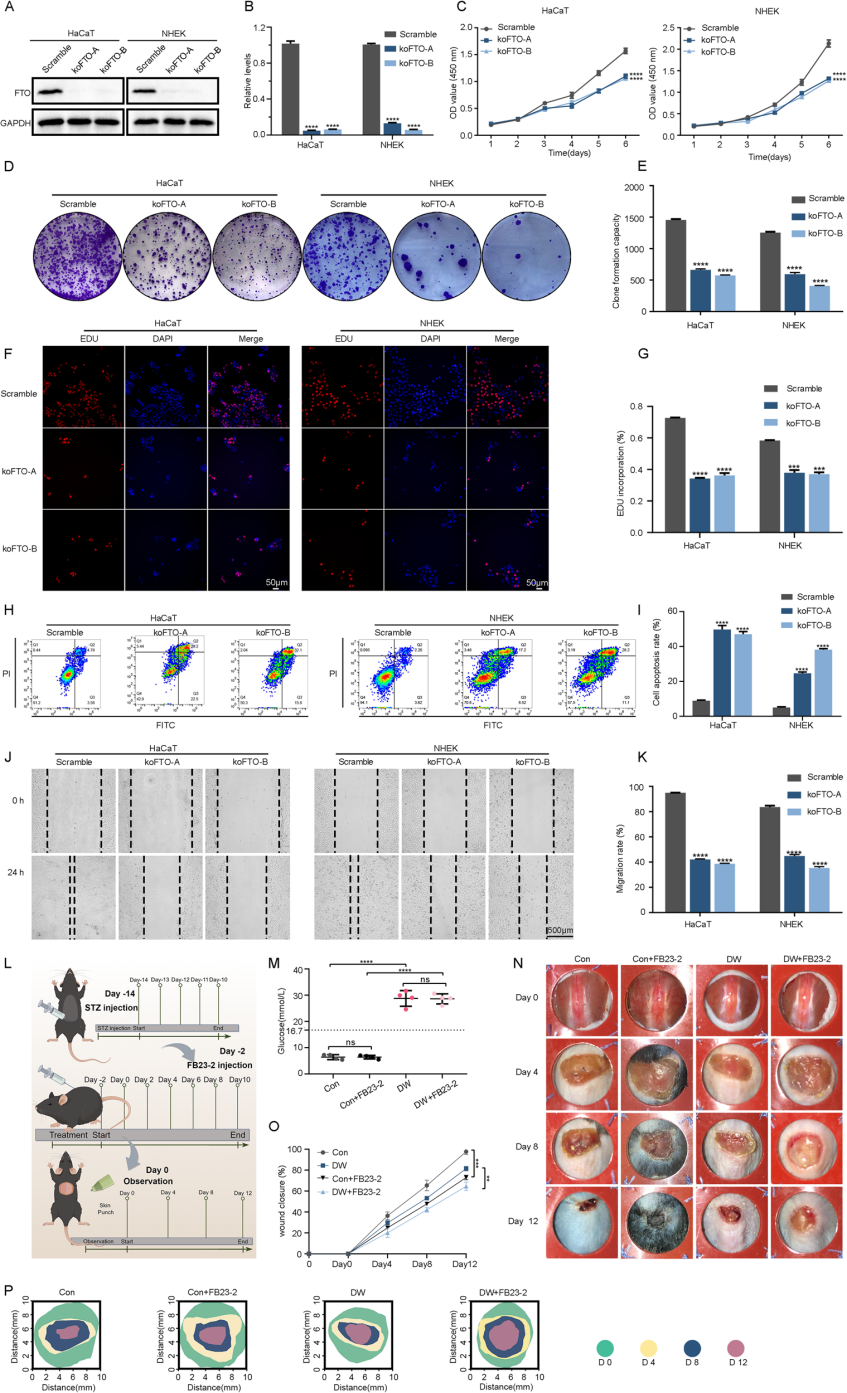
Silencing FTO inhibited the proliferation and migration of keratinocytes. **A, B** Immunoblot analysis to verify the decrease in FTO protein levels in scramble and FTO knockout HaCaT and NHEK cells. The quantification results of FTO protein expression are shown (*n* = 3). **C**–**E** Silencing FTO expression decreased proliferation, as reflected by the CCK-8 and colony formation assay results. Semi-quantitative analysis of colony formation assays is shown (*n* = 3). **F, G** The EdU incorporation assay was conducted in scramble and FTO knockout HaCaT and NHEK cells to detect cell proliferation, and the EdU positive cells were quantitated (*n* = 3; Scale bar, 50 μm). **H, I** Flow cytometry analysis was used to examine cell apoptosis rates of the scramble and related FTO knockout cells (HaCaT and NHEK, *n* = 3). **J, K** HaCaT and NHEK cells were infected with scramble or two different FTO knockout viruses, and images were captured at 0 h and 24 h after the scratch. Semi-quantitative analysis of wound-healing assays is shown (*n* = 3; Scale bar, 500 μm). **L** Illustration of the experimental timeline for the STZ and FB23-2 injections and wound healing models of mice. **M** Measurements of blood glucose levels in control and STZ-treated mice. **N**–**P** Representative images of cutaneous wounds of control and diabetic mice with or without FB23-2 injection on days 0, 4, 8, and 12 after wound generation by surgical excision. Rates of wound closure were quantified using ImageJ software and expressed as the percentage of closed wound area (*n* = 5 per group). The data are presented as the mean ± SD or mean ± SEM (**B, C, E, G, I, K, M, N**), and *P*-values of all data by a two-tailed unpaired t-test are indicated. *P < 0.05, **P < 0.01, ***P < 0.001, ****P < 0.0001.

To clarify the functional significance of FTO downregulation in thinner diabetic epidermis and slow wound healing, we then assessed the effects of FTO-knockout (KO) in HaCaT and NHEK cells. Firstly, the knockout efficiency of FTO was validated (Fig. 2A, B). FTO-deletion caused a decrease in cell growth (Fig. 2C), clonogenic growth (Fig. 2D, E), and the percentages of 5-Ethynyl-2’-deoxyuridine (Edu)-positive cells (Fig. 2F, G). Lowered FTO expression of HaCaT and NHEK cells led to increased apoptosis (Fig. 2H, I). In addition, the scratch assay showed significantly migration delay in FTO-KO HaCaT and NHEK cells (Fig. 2J, K). To investigate the in vivo role of FTO in wound healing under chronic hyperglycemia conditions, we induced diabetic models in C57BL/6 WT mice with streptozotocin (STZ) (Fig. 2L). The blood glucose level was obviously higher in STZ-induced diabetic mice than in the normal controls (Fig. 2M). Notably, wound closure in diabetic mice was significantly delayed compared to that in the controls (Fig. 2N–P). To examine how FTO influences wound healing, FTO inhibitors (FB23-2)-based treatment were used to decrease demethylase activity mediated by FTO in the skin (Fig. 2L). Compared to control or diabetic mice without treatment, the groups injected with FB23-2 showed delayed wound healing (Fig. 2N-P). To further verify the above results, we repeated the above experiments in FTO-knockdown HaCaT and NHEK cells, and the results were consistent with the above experiments (SFig. 2).

### Elevated FTO expression contributes to the development of keratinocytes

To further reveal the role of FTO in regulating keratinocytes with high-glucose treatment, FTO-overexpression was achieved by vector transfection in HaCaT and NHEK cells (SFig. 3A, B). Control and FTO-overexpressing HaCaT and NHEK cells were treated with NG or high-glucose (30 mM) medium. The results showed that high-glucose decreased proliferation and migration of HaCaT and NHEK cells, which were rescued by overexpression of FTO (SFig. 3C-I). We also established diabetic wound models with C57BL/6 mice (SFig. 3J, K). To explore the functional roles of FTO in wound healing, we performed the in vivo experiment by injecting the FTO-overexpression plasmid and the control plasmid into the skin of mice. Remarkably, the wound healing efficiency of DW-oeFTO mice was strikingly increased compared to control mice (SFig. 3L-N). Together, these results suggested that FTO had a remarkable biological effect on keratinocytes, including proliferation and migration.

### High-glucose induced autophagy through FTO downregulation in keratinocytes

**Fig. 3:**
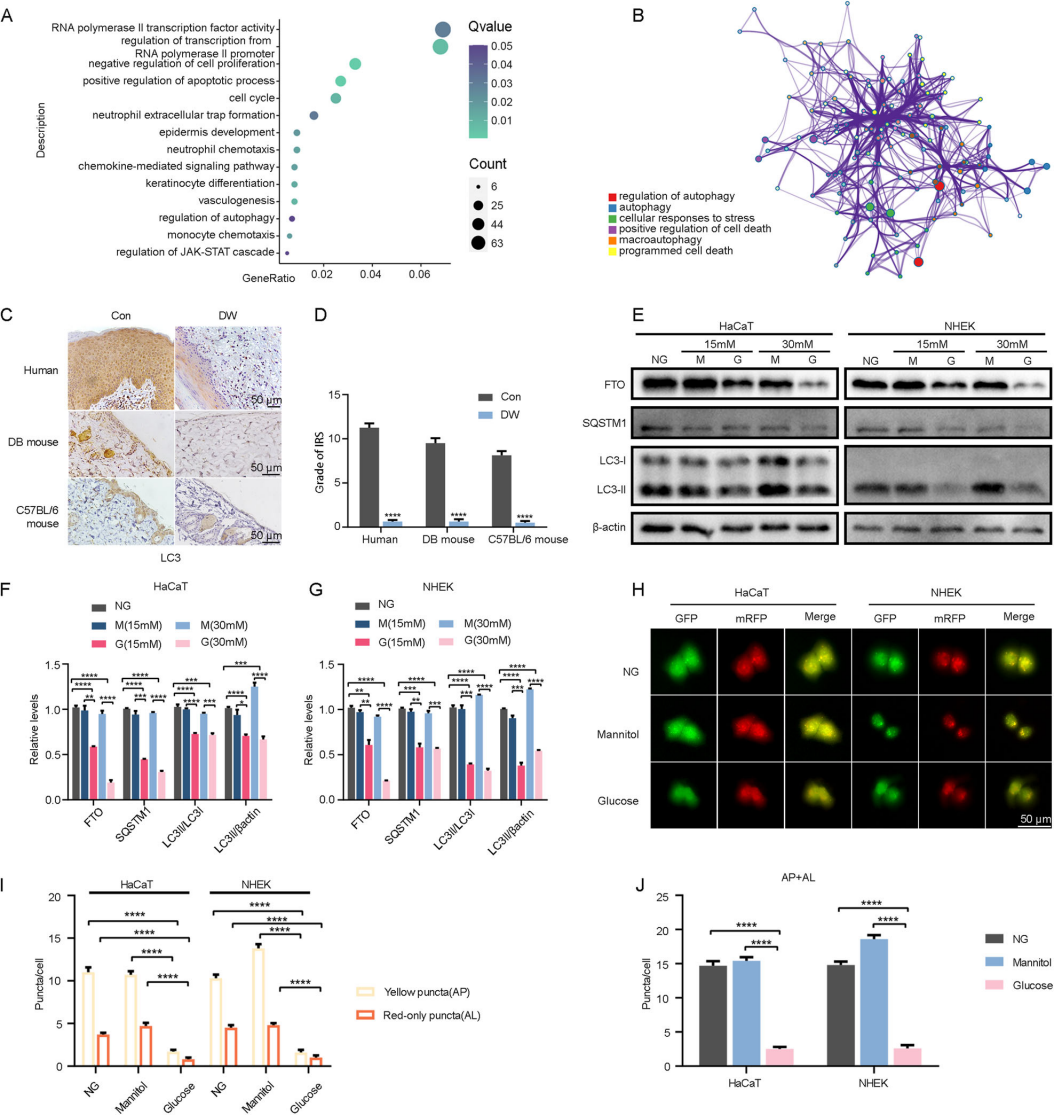
High glucose induced downregulation of FTO and impairment of autophagic flux in keratinocytes. **A** Functional enrichment by differentially expressed genes (DEGs) between scramble and knockout FTO HaCaT cells (*n* = 3). **B** The PPI networks were constructed by the STRING database for modules with a threshold value > 0.4. **C, D** IHC analysis of LC3 in the epidermis of DW and the control group in humans, DB-strain mice, and C57BL/6 mice (Scale bar, 50 μm). Quantitative analysis of FTO protein (brown color) levels in the epidermis was performed according to the IRS (*n* = 5). **E**–**G** Western blot analysis of FTO, SQSTM1, LC3I, and LC3II expression levels in HaCaT and NHEK cells treated with NG (normal glucose: 5.6 mM of glucose), 15 mM G (mid-high glucose: 15 mM of glucose), 30 mM G (high glucose: 30 mM of glucose), 15 mM M (15 mM: 5.6 mM of glucose + 9.4 mM of mannitol), and 30 mM M (5.6 mM of glucose + 24.4 mM of mannitol) for 72 h. Quantification results of protein expression are shown (*n* = 3). **H**–**J** The HaCaT and NHEK cells were infected with adenovirus harboring tandem fluorescent mRFP-GFP-LC3 for 24 h, followed by treatment with NG, 15 mM M, 15 mM G, 30 mM M, and 30 mM G for an additional 72 h. Representative images of the HaCaT and NHEK cells expressing mRFP-GFP-LC3 are shown. Green: GFP puncta; red: mRFP puncta. Semi-quantitative analysis of autophagosomes (AP, yellow puncta in merged images) and autolysosomes (AL, red-only puncta in merged images, *n* = 10 randomly selected conditions from 3 independent experiments, Scale bar, 50 μm). The data are presented as the mean ± SD or mean ± SEM (**D, F, G, I, J**), and *P* values of all data by a two-tailed unpaired t-test are indicated. *P < 0.05, **P < 0.01, ***P < 0.001, ****P < 0.0001.

We proceeded to examine the specific mechanism by which FTO regulated biological and physiological functions of keratinocytes. The mRNA profiles of scramble and FTO-knockout HaCaT cells were sequenced using an Illumina Novaseq™ 6000 system. We analyzed the dataset, and functional enrichment analysis demonstrated that DEGs were significantly enriched in m^6^A methylation and autophagy (Fig. 3A, B). This finding indicated that FTO affected keratinocyte biological functions by modulating autophagy. To validate this, we performed IHC and found that the expression level of LC3 was obviously reduced both in the diabetic epidermis of humans and mice (Fig. 3C, D). To examine the impact of high-glucose on autophagy, HaCaT and NHEK cells were treated with NG, different glucose concentrations, and mannitol. We noted that SQSTM1 expression, the LC3II/LC3I ratio, and the LC3II/β-actin ratio were significantly decreased in high-glucose treatment accompanied by the reduction of FTO (Fig. 3E–G). Autophagic flux was monitored by transfection with mRFP-GFP-LC3. Compared with the NG and the mannitol control, high-glucose treatment showed the decrease in red (autolysosomes) and yellow (autophagosomes) fluorescent puncta, indicating the decrease in either autolysosomes or autophagosomes, which reflected the inhibition of autophagy (Fig. 3H–J) [24]. Taken together, these results indicated that hyperglycemia induced inhibition of autophagy in keratinocytes.

### FTO regulates autophagy in the high-glucose environment of keratinocytes

**Fig. 4:**
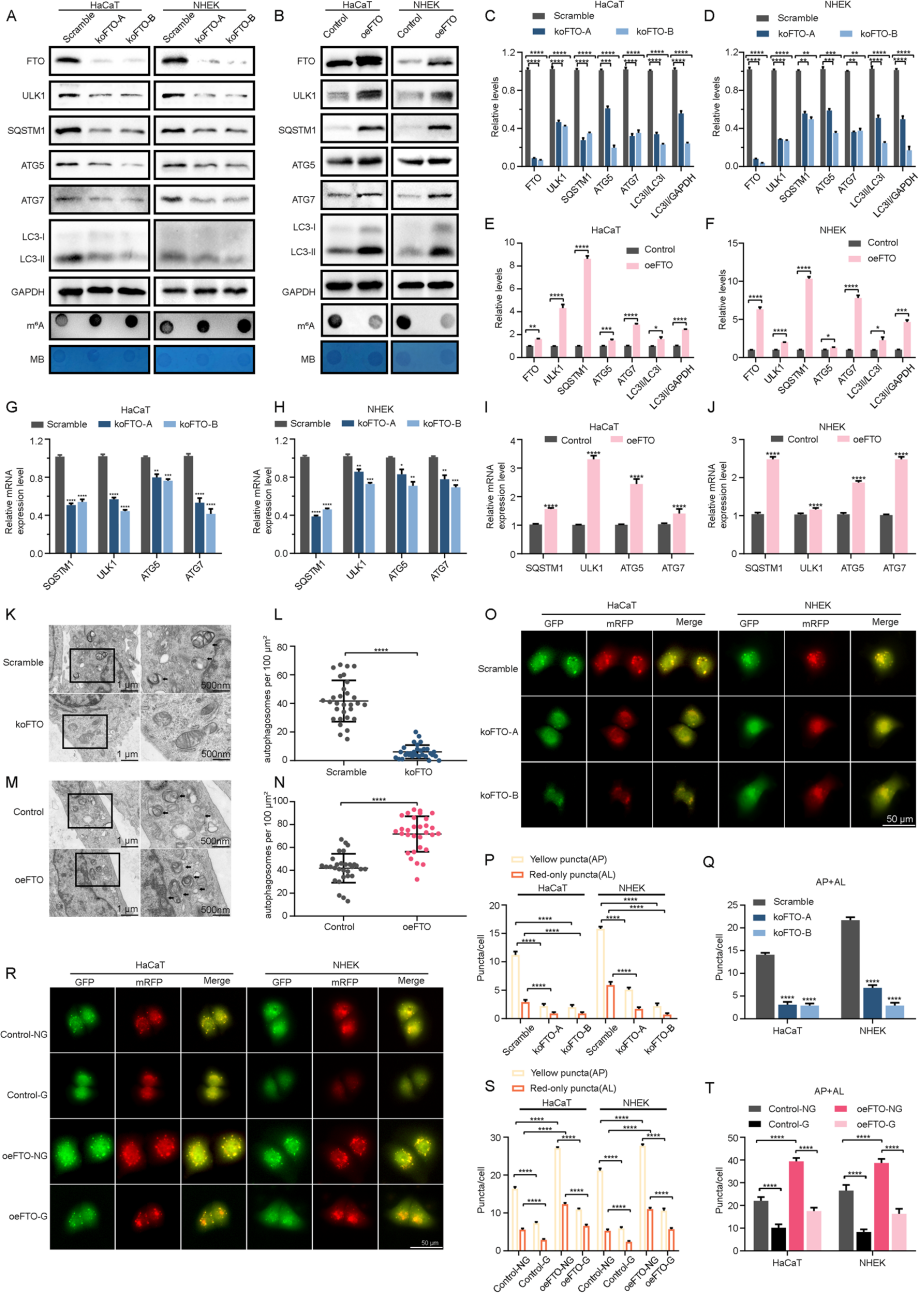
FTO regulated autophagic flux by modulating m^6^A levels. **A, C, D** Immunoblot analysis of FTO, ULK1, SQSTM1, ATG5, ATG7, LC3I, LC3II, and m6A dot blot assay in scramble and FTO knockout HaCaT and NHEK cells. Methylene blue (MB) staining was used as a loading control. Quantification results of FTO, ULK1, SQSTM1, ATG5, ATG7 protein expression, LC3II/LC3I ratio, and LC3II/GAPDH ratio levels are shown (*n* = 3). **B, E, F** Immunoblot analysis of FTO, ULK1, SQSTM1, ATG5, ATG7, LC3I, LC3II, and m6A dot blot assay in control and FTO-overexpressing HaCaT and NHEK cells. MB staining was used as a loading control. Quantification results of FTO, ULK1, SQSTM1, ATG5, ATG7 protein expression, LC3II/LC3I ratio, and LC3II/GAPDH ratio levels are shown (*n* = 3). **G, H** RT-qPCR analysis of autophagy-related mRNA expression levels in control and FTO knockout HaCaT and NHEK cells (*n* = 3). **I, J** RT-qPCR analysis of autophagy-related mRNA expression levels in control and FTO-overexpressing HaCaT and NHEK cells. **K, L** TEM analysis of scramble and FTO knockout HaCaT and NHEK cells (Arrows indicate autolysosomes and/or lysosomes. Scale bar, 1.0 μm and 500 nm). **M, N** TEM analysis of control and FTO knockout HaCaT and NHEK cells (Arrows indicate autolysosomes and/or lysosomes. Scale bar, 1.0 μm and 500 nm). **O**–**Q** The HaCaT and NHEK cells were infected with adenovirus harboring tandem fluorescent mRFP-GFP-LC3 for 24 h, followed by transfection with sgRNAs in normal-glucose culture medium. Representative images of the HaCaT and NHEK cells expressing mRFP-GFP-LC3 are shown. Green: GFP puncta; red: mRFP puncta. Semi-quantitative analysis of autophagosomes (AP, yellow puncta in merged images) and autolysosomes (AL, red-only puncta in merged images, *n* = 10 randomly selected conditions from 3 independent experiments, Scale bar, 50 μm). **R**–**T** The HaCaT and NHEK cells overexpressing FTO and the control were infected with adenovirus harboring tandem fluorescent mRFP-GFP-LC3 for 24 h, followed by treatment with normal glucose or high glucose (30 mM) for an additional 72 h. Representative images of the HaCaT and NHEK cells expressing mRFP-GFP-LC3 are shown. Green: GFP puncta, red: mRFP puncta. Semi-quantitative analysis of autophagosomes (AP, yellow puncta in merged images) and autolysosomes (AL, red-only puncta in merged images, *n* = 10 randomly selected conditions from 3 independent experiments, Scale bar, 50 μm). The data are presented as the mean ± SD or mean ± SEM (**A, B, C, D, G, H, I, J, L, N, P, Q, S, T**), and *P*-values of all data by a two-tailed unpaired t-test are indicated. *P < 0.05, **P < 0.01, ***P < 0.001, ****P < 0.0001.

**Fig. 5:**
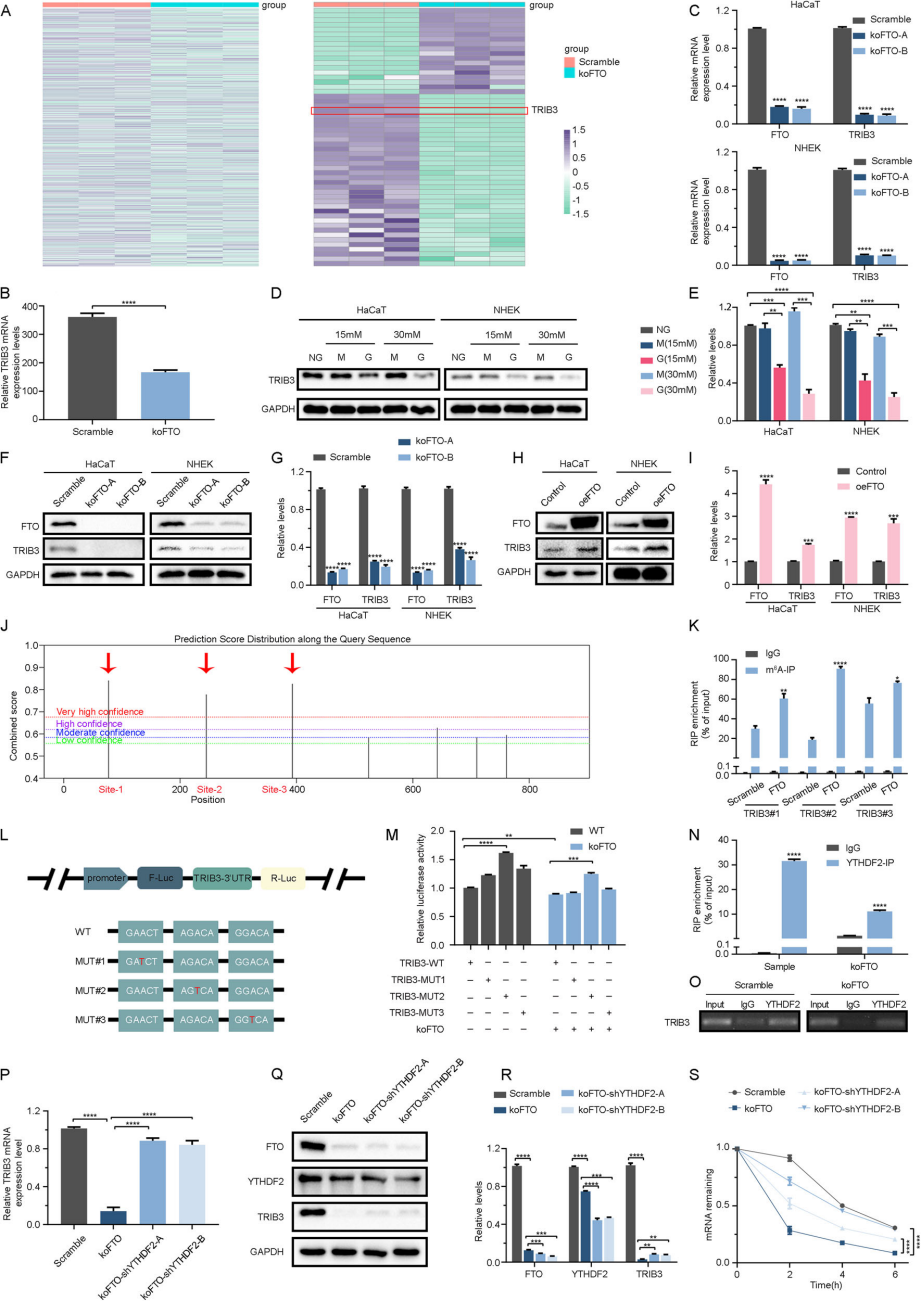
FTO regulated TRIB3 mRNA levels in an m^6^A-YTHDF2-dependent manner. **A** Heatmaps showed DEGs and autophagy-related DEGs in scramble and FTO knockout HaCaT cells. **B** Expression levels of TRIB3 in the mRNA profiles of scramble and FTO knockout HaCaT cells. **C** The TRIB3 expression was validated by RT-qPCR in scramble and FTO knockout HaCaT cells. **D, E** Western blot analysis of TRIB3 expression levels in HaCaT and NHEK cells treated with NG (normal glucose: 5.6 mM of glucose), 15 mM G (mid-high glucose: 15 mM of glucose), 30 mM G (high glucose: 30 mM of glucose), 15 mM M (15 mM: 5.6 mM of glucose + 9.4 mM of mannitol), and 30 mM M (5.6 mM of glucose + 24.4 mM of mannitol) for 72 h. Quantification results of TRIB3 protein expression levels are shown (*n* = 3). **F,G** FTO knockdown decreased TRIB3 protein levels. **H, I** FTO overexpression increased TRIB3 protein levels. **J** Integrative Genomics Viewer (IGV) analysis showed that FTO attenuation increased m^6^A modification levels of TRIB3 mRNA. **K** MeRIP-qPCR analysis confirmed that the m^6^A modification of TRIB3 mRNA was enriched upon FTO knockdown. **L** Wild-type or m^6^A consensus sequence mutant TRIB3 3’UTR was fused with firefly luciferase reporter. Mutation of m^6^A consensus sequences was generated by replacing adenosine with thymine. **M** Relative luciferase activity of the wild-type and mutant TRIB3 3’UTR reporter vectors. **N, O** YTHDF2 was immunoprecipitated and then subjected to RT-qPCR to assess TRIB3 transcript levels. Agarose gel electrophoresis of RT-qPCR amplification products to verify immunoprecipitated results. **P** RT-qPCR analysis of TRIB3 mRNA levels (scramble and YTHDF2 knockdown) in the absence or presence of FTO knockdown. **Q, R** Immunoblotting assay of YTHDF2 protein levels in HaCaT cells with scramble, FTO knockdown, and YTHDF2 knockdown. **S** RT-qPCR analysis of TRIB3 mRNA levels (scramble and YTHDF2 knockdown) in the absence or presence of FTO knockdown after actinomycin D treatment. The data are presented as the mean ± SD or mean ± SEM (**B, C, E, G, I, K, M, N, P, R, S**), and *P*-values of all data by a two-tailed unpaired t-test are indicated. *P < 0.05, **P < 0.01, ***P < 0.001, ****P < 0.0001.

To investigate the function of FTO in autophagy, FTO-knockout in HaCaT and NHEK cells using CRISPR (Fig. 4A). Following FTO-knockout, we observed that the downregulation of SQSTM1, ULK1, ATG5, and ATG7 protein levels (Fig. 4C, D). FTO-depletion resulted in increased m^6^A levels and inhibited autophagy. Subsequently, FTO-overexpression in HaCaT and NHEK cells led to opposite results (Fig. 4B, E, F). As shown in Fig. 4G–J, mRNA levels of autophagy-related genes examined were consistent with the protein expression results. Based on the above observations, transmission electron microscopy (TEM) was applied to visualize the ultrastructures of autophagy. It was shown that FTO-depleted cells inhibited autophagy, while FTO-overexpressing cells promoted autophagy (Fig. 4K–N). Furthermore, knockout of FTO HaCaT and NHEK cells inhibited autophagy by blocking autophagosome formation (Fig. 4O–Q). Consistently, high-glucose induced autophagy inhibition, which could be rescued by FTO-overexpression in HaCaT and NHEK cells (Fig. 5R–T).

### Identification of TRIB3 as the functional target of FTO

We further mined our mRNA profiles of scramble and knockout HaCaT cells, heatmaps were performed on the DEGs and autophagy-related DEGs (Fig. 5A). In these autophagy-related DEGs, we found that TRIB3 was decreased in FTO-knockout HaCaT and NHEK cells as compared with scramble (Fig. 5B), and RT-qPCR verified the same trend (Fig. 5C). Meanwhile, keratinocytes with high-glucose treatment downregulated TRIB3, and decreased FTO expression contributed to the reduction of TRIB3 expression, while overexpressing FTO upregulated TRIB3 (Fig. 5D–I). To ascertain whether FTO can directly affect specific m^6^A methylation sites of TRIB3, the m^6^A modification site predictor (https://www.cuilab.cn/sramp) was performed to screen for the potential m^6^A sites of TRIB3 mRNA, including TRIB3 site-1, site-2, and site-3 (Fig. 5J). MeRIP-qPCR showed that FTO-knockout significantly facilitated m^6^A levels in TRIB3 mRNA compared to the IgG-RIP group (Fig. 5K). Our study further proved the effect of the m^6^A modification on TRIB3 mRNA by replacing N^6^-methylated adenosine (A) with thymine (T) at the three predicted m^6^A sites and cloning them into the dual-luciferase reporter construct pmirGLO to generate reporter vectors (Fig. 5L). Then, luciferase reporter assays were performed in Human Embryonic Kidney (293 T) cells that were transfected with the wild-type and three mutant dual-luciferase reporter plasmids of TRIB3. Notably, luciferase activity was significantly inhibited in FTO-knockout cells. TRIB3-MUT2 almost abolished this induction, showing that FTO-associated m^6^A modification of MUT2 modulated TRIB3 expression (Fig. 5M).

The YTHDF family is thought to be the major component that recognizes m^6^A-modified transcripts [6] and m^6^A sites by recruiting one or more of the YTHDF proteins to the mRNA. We further verified the reader protein of methylated TRIB3 mRNA. Through RNA-immunoprecipitation-qPCR (RIP-qPCR), we observed that mRNA can be recognized and bound by YTHDF2 in HaCaT, and agarose gel electrophoresis of the PCR products further confirmed the above results (Fig. 5N, O). YTHDF2 was enriched on TRIB3 mRNA, while FTO-knockout significantly reduced the amount of YTHDF2 bound to TRIB3 mRNA. In addition, we knocked down YTHDF2 in FTO-depleted HaCaT cells and discovered partially restored TRIB3 expression (Fig. 5P–R). These results confirmed that TRIB3 was a target of YTHDF2. YTHDF2 knockdown not only enhanced the levels and stability of TRIB3 mRNA but also abrogated their reduction under FTO-knockout conditions (Fig. 5S). Thus, these results suggest that YTHDF2 regulates TRIB3 dependent on the binding of YTHDF2 to m^6^A-modified mRNA.

### The effects of FTO inhibition are reversed by overexpression of TRIB3 in vitro and in vivo

**Fig. 6:**
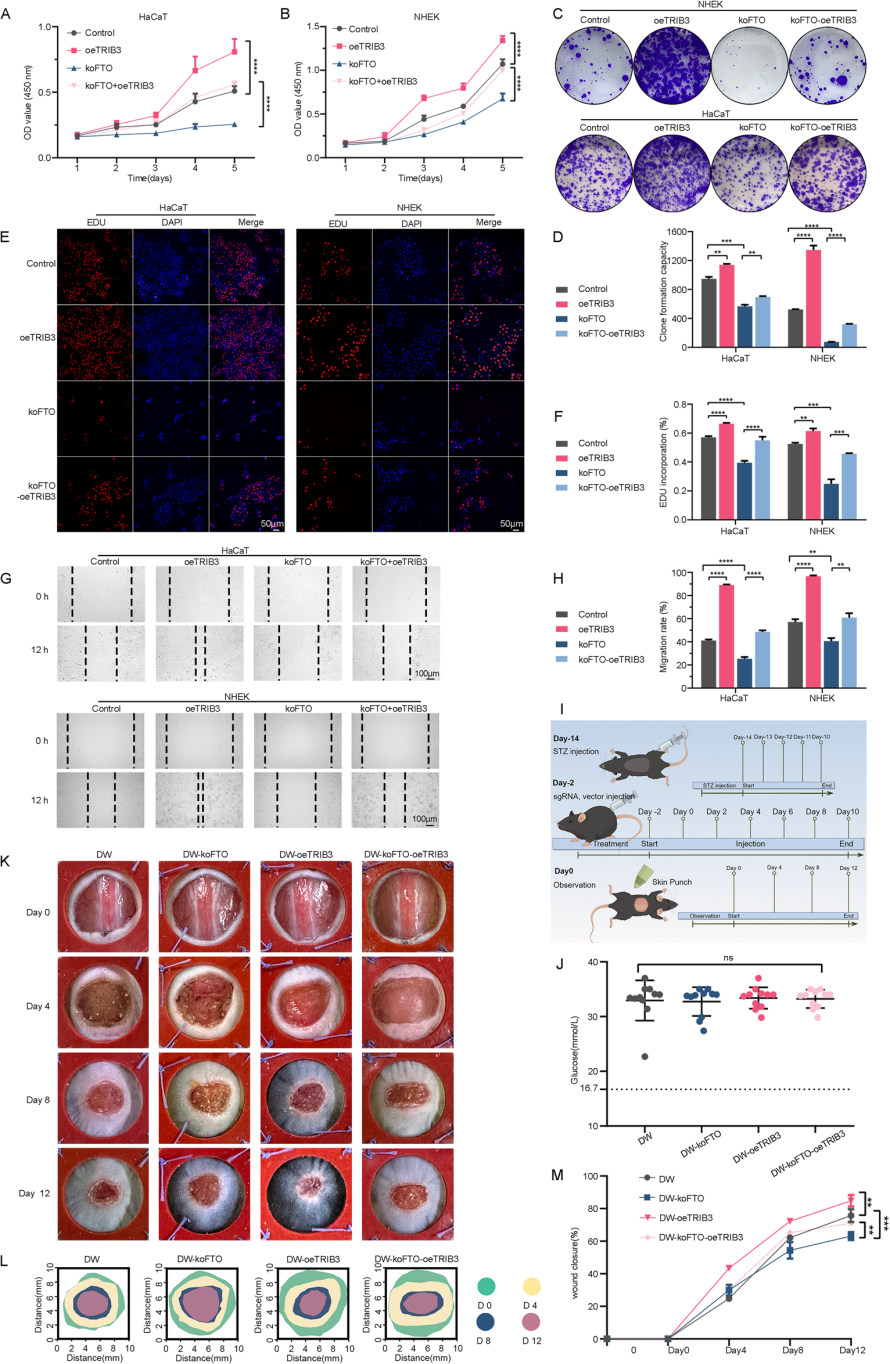
FTO influenced proliferation and migration by affecting the expression of TRIB3. **A-D** Overexpression of TRIB3 rescued the proliferative activity of HaCaT and NHEK cells with FTO knockout, as reflected by CCK-8 and colony formation assays. Semi-quantitative analysis of colony formation assays is shown (*n* = 3). **E, F** The Edu incorporation assays revealed that FTO significantly inhibits proliferation of keratinocytes, which can be rescued by overexpression of TRIB3. **G, H** Overexpression of TRIB3 during FTO knockdown partially rescued the migration of keratinocytes. Images were captured at 0 h and 12 h after the scratch. Semi-quantitative analysis of wound-healing assays is shown (*n* = 3). **I** Timeline of in vivo experiments for STZ injections, sgRNA and vector injections, and observation of wound healing models in mice. **J** Measurements of blood glucose levels in STZ-treated mice. **K-M** Representative images of cutaneous wounds of diabetic mice, diabetic mice with koFTO injections, diabetic mice with oeTRIB3 injections, and diabetic mice with koFTO and oeTRIB3 injections on days 0, 4, 8, and 12 after wound generation by surgical excision. Rates of wound closure were quantified using ImageJ software and expressed as the percentage of closed wound area (*n* = 5 per group). The data are presented as the mean ± SD or mean ± SEM (**A, B, D, F, H, J, M**), and *P*-values of all data by a two-tailed unpaired t-test are indicated. *P < 0.05, **P < 0.01, ***P < 0.001, ****P < 0.0001.

We explored whether TRIB3 mediated the function of FTO in keratinocyte proliferation and migration, the control and TRIB3 overexpression vectors were transfected into FTO-knockout HaCaT and NHEK cells. We found that TRIB3 overexpression not only increased HaCaT and NHEK cells in both proliferation and migration but also abrogated the reduction under FTO-knockout (Fig. 6A–H). To support in vivo evidence for the functional role of FTO and TRIB3 in wound closure under consistent hyperglycemia, we established diabetic wound models with C57BL/6 mice (Fig. 6I). The levels of blood glucose remained above 16.7 mM for 4 weeks (Fig. 6J). The wound closure rate in DW-oeTRIB3 mice was remarkably enhanced compared with diabetic mice and alleviated wound healing delay in the skin of mice with FTO-knockout injection (Fig. 6K–M).

### FTO induces TRIB3 interaction with SQSTM1 to hinder autophagic degradation

**Fig. 7:**
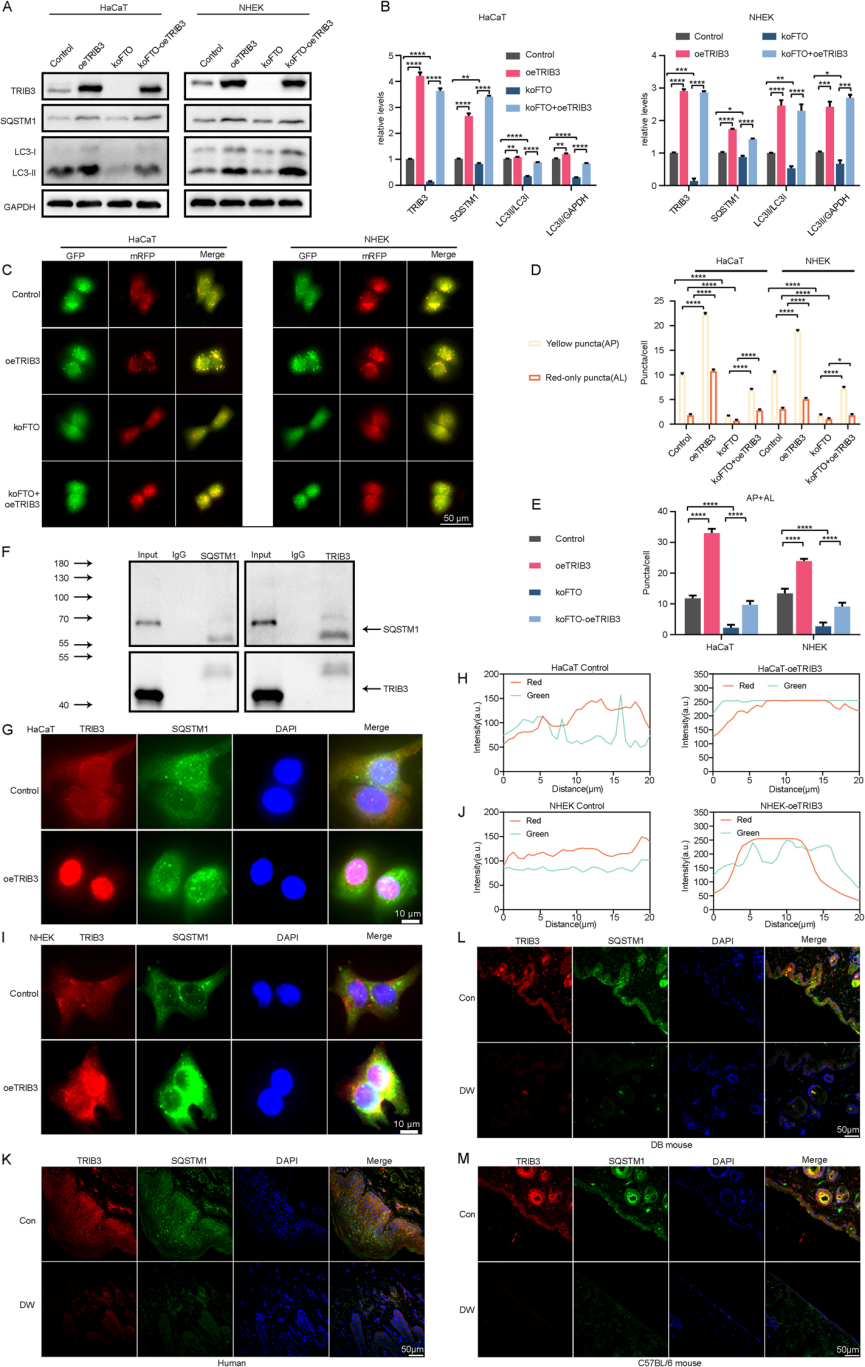
FTO regulated autophagy by targeting TRIB3, which inhibited autophagic degradation by interacting with SQSTM1. **A, B** TRIB3, SQSTM1, LC3I, and LC3II protein levels were measured by western blot in HaCaT and NHEK cells transfected with lentiviruses carrying koFTO and/or oeTRIB3 (*n* = 3). **C–E** The HaCaT and NHEK cells were infected with adenovirus harboring tandem fluorescent mRFP-GFP-LC3 for 24 h, followed by transfection with oeTRIB3, koFTO, and koFTO with oeTRIB3 in normal-glucose culture medium. Representative images of the HaCaT and NHEK cells expressing mRFP-GFP-LC3 are shown. Green: GFP puncta; red: mRFP puncta. Semi-quantitative analysis of autophagosomes (AP, yellow puncta in merged images) and autolysosomes (AL, red-only puncta in merged images, n = 10 randomly selected conditions from 3 independent experiments, Scale bar, 50 μm). **F** The TRIB3-SQSTM1 interaction was evaluated by an IP assay in TRIB3-overexpressing HaCaT cells (*n* = 3). **G, I** Colocalization of TRIB3 and SQSTM1 was detected by immunostaining in control and TRIB3-overexpressing HaCaT and NHEK cells. **H, J** Line chart of fluorescence signal positioning analysis (*n* = 3; Scale bar, 10 μm). **K–M** Expression of TRIB3 and colocalization of TRIB3 with SQSTM1 in Con and DW tissues were detected by immunostaining. (*n* = 5 per group; Scale bar, 50 μm). The data are presented as the mean ± SD or mean ± SEM (**B, D, E**), and *P*-values of all data by a two-tailed unpaired t-test are indicated. *P < 0.05, **P < 0.01, ***P < 0.001, ****P < 0.0001.

To identify the correlation between FTO-mediated TRIB3 and autophagic activity, we analyzed the expression levels of TRIB3 and autophagy-related proteins. Knockout of FTO decreased the expression of SQSTM1 expression, the LC3II/LC3I ratio, and the LC3II/β-actin ratio, whereas overexpression of TRIB3 rescued this decrease (Fig. 7A, B). To investigate the role of TRIB3 in autophagy, HaCaT and NHEK cells were transfected with mRFP-GFP-LC3. Elevated TRIB3 expression increased the numbers of autophagosomes as well as autolysosomes and reversed the inhibition of autophagy in FTO-knockout (Fig. 7C–E). Previous studies showed that TRIB3 directly interacts with SQSTM1 and interferes with its binding to LC3, which suppresses autophagic flux [25]. In keratinocytes, we found that TRIB3 was co-immunoprecipitated with SQSTM1, which was consistent with previous research (Fig. 7F). TRIB3 and SQSTM1 showed colocalization, and TRIB3 overexpression increased the colocalization of TRIB3 with SQSTM1 (Fig. 7G–J). Moreover, we observed a positive association between TRIB3 and SQSTM1 expression in normal and diabetic epidermis. Diabetic epidermis decreased expression of TRIB3, as well as reduced colocalization of TRIB3 with SQSTM1, compared with normal tissue in human, type I and II diabetic mice. (Fig. 7K–M). Line chart of fluorescence signal positioning analysis in human and mouse tissues in SFig. 4A-F.

## Discussion

**Fig. 8:**
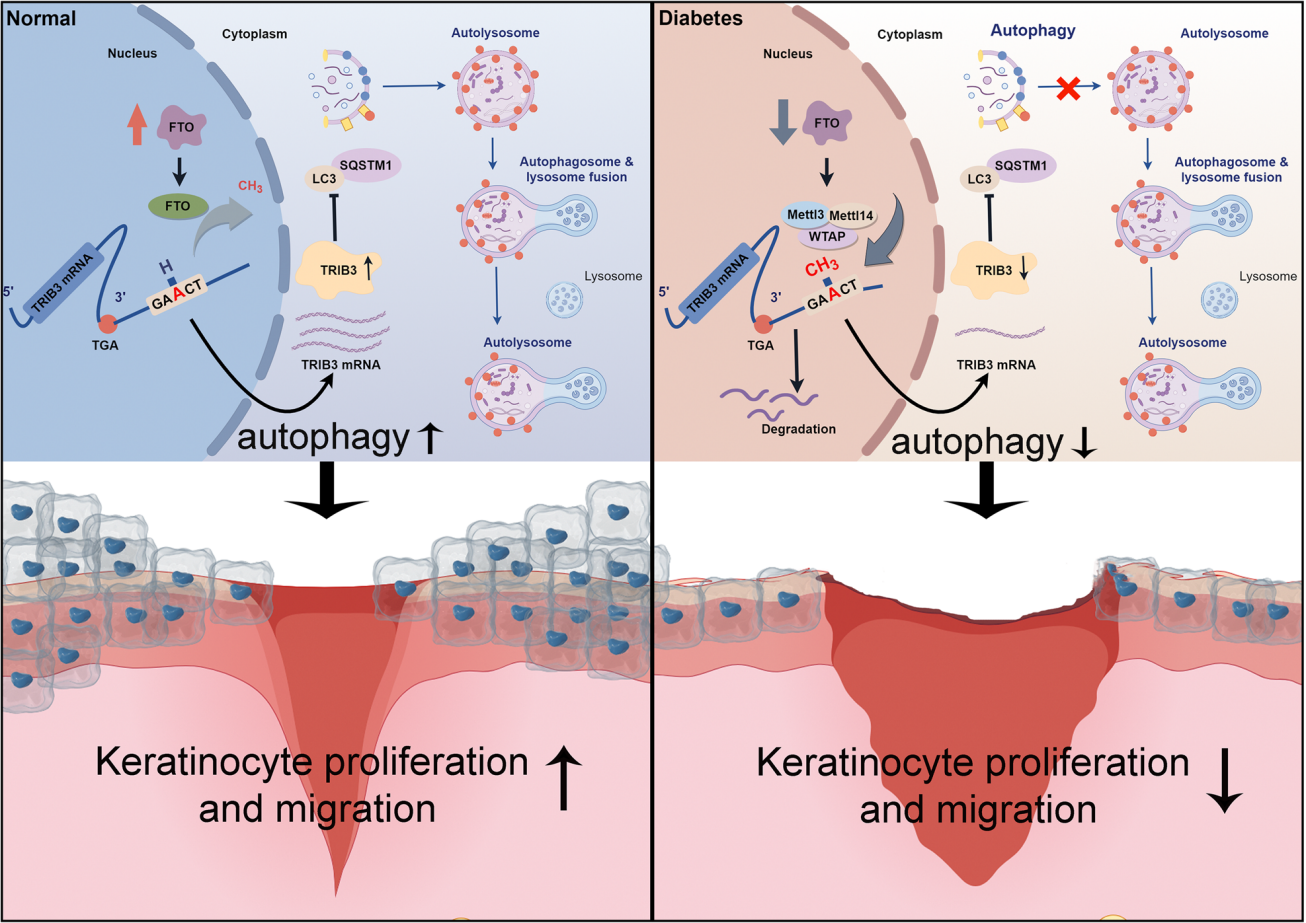
Proposed working models of FTO in the regulation of autophagy in keratinocytes of diabetic skin. The expression of TRIB3 was regulated by FTO, and it interacted and cooperated with SQSTM1 to induce autophagy in keratinocytes. In diabetes, downregulation of FTO induced decreased expression levels of TRIB3 via acceleration of TRIB3 mRNA decay, which led to the inhibition of autophagy and the impairment of keratinocyte migration, eventually resulting in delayed wound healing.

Diabetes has a pronounced negative effect on wound healing. Foot ulcers in DM patients remain the main cause of morbidity and mortality in patients with diabetes [2, 26]. The process of wound healing is multicellular and multistep, and the epidermis covers the dermis, with the epidermis acting as a protective barrier against pathogens and controlling the release of water in the dermis [27, 28]. The epidermis contains approximately 90% keratinocytes. Recently, the crucial role of keratinocytes in cutaneous wound healing has been explored. Keratinocytes participate in the healing process by covering dermal surfaces, restoring mucosal homeostasis, and dampening the inflammatory response [29, 30]. The physiological dysfunctions of epidermal cells in high-glucose environments include prolonged inflammation, impaired migration and proliferation, and delayed wound healing [31]. M^6^A modifications are shown to play a regulatory role in controlling skin biology and disease pathogenesis [32, 33]. However, the role of m^6^A in biological functions of the skin epidermis caused by high-glucose environments remains unknown. Here we discovered that high-glucose exposure downregulated FTO and upregulated m^6^A enrichment in keratinocytes, decreasing the proliferation and migration of keratinocytes. At the molecular level, downregulation of FTO was found to be responsible for the decrease of TRIB3 and inhibited proliferation and migration by inhibiting autophagy in keratinocytes with high-glucose treatment. Moreover, we further confirmed the functional role of FTO-mediated m^6^A modification of TRIB3 mRNA in keratinocytes and subsequently enhanced its stability in a YTHDF2-dependent manner. In summary, these findings establish m^6^A eraser FTO as an epitranscriptomic mechanism in the biological function of the diabetic epidermis (Fig. 8).

Our study benefited genome-wide functional screens of m^6^A biology function in the epidermal barrier [34]. These findings indicated a broad effect of m^6^A modifications in mediating keratinocyte proliferation and differentiation, epidermal barrier development, and wound healing processes [33]. In this research, we found that the m^6^A modification was upregulated in keratinocytes with high-glucose treatment using dot-blotting. After mapping the expression of m^6^A in the epidermis of diabetic patients, type I and II diabetic mice, we further proposed that the increased m^6^A level may be ascribed to the downregulation of m^6^A demethylase FTO in keratinocytes validated using immunofluorescence staining. Here, we presented a novel regulatory mechanism by which FTO modulated keratinocyte proliferation and migration by removing m^6^A modifications. These results were further confirmed in different keratinocyte cell lines. Epidermis thinning in diabetes can be attributed to cell dysfunction and autophagy impairment caused by a decrease in FTO in keratinocytes. These findings highlight the crucial function of the m^6^A regulator in regulating autophagy, the dysfunction of which may result in delayed diabetic wound healing.

Autophagy plays distinct regulatory roles in multiple cell types of diabetic skin [35, 36]. The findings of the recent study found that autophagy was inhibited in keratinocytes with high-glucose treatment [19]. In the present study, we showed that the downregulation of FTO increased the m^6^A modifications of TRIB3 and inhibited autophagy in diabetic keratinocytes. Bioinformatic analyses combining mRNA and protein expression of TRIB3 are downregulated in FTO knockout HaCaT samples, suggesting that TRIB3 may function as a downstream gene of FTO. As a scaffolding protein in protein complex assembly, the pseudokinase TRIB3 coordinates crucial cellular processes, such as glucose and lipid metabolism, adipocyte differentiation, and apoptosis [37, 38]. TRIB3 promotes chronic inflammation by interacting with intracellular signaling and functional proteins. Research suggests that TRIB3 interacts with different regulatory genes to govern several diseases, including diabetes, hepatitis, and tissue fibrosis [39, 40]. Much research has found a relationship between TRIB3 and SQSTM1, which may be a key component of autophagy. This TRIB3-SQSTM1 interaction abolishes the binding of LC3 to SQSTM1, thus inducing the blockage of autophagic flux [41, 42]. Furthermore, TRIB3 played crucial roles in keratinocyte development and progression by facilitating autophagy, which regulated wound closure. Further in vivo and in vitro evidence was proposed for the potential mechanistic roles of FTO in regulating TRIB3 and autophagy, as well as its functional role in diabetic wound healing. Research on m^6^A modification and diabetic wounds is still an emerging field, while the roles of FTO in diabetic wound healing have not been studied. TRIB3 was also found to be a new FTO target gene, first used in wound studies. Hydrogels with viral vectors may be more suitable for the diabetic microenvironment, which serves as a target for the next stage of our research [43].

The current research investigated that downregulation of FTO played key roles in suppressing keratinocyte autophagy under both acute and chronic hyperglycemia environments, implying that FTO can be used as a biomarker and potential therapeutic target for diabetic wounds, and modulated m^6^A modification in TRIB3 mRNA levels via an m^6^A-YTHDF2-dependent manner. Here, our study verifies the pro-healing activity of FTO in vitro and in vivo, and TRIB3, a downstream target of FTO, directly interacts with SQSTM1 which regulates autophagy. We also revealed that the overexpression of TRIB3 reversed the trends of FTO downregulation in diabetic wounds. FTO mediated m^6^A in the 3’UTR of TRIB3 mRNA via an m^6^A-YTHDF2-dependent manner and modulates expression in post-transcriptional regulation. TRIB3 acted as autophagy-regulator and alleviated the suppression of proliferation and migration in FTO-KO keratinocytes. Therefore, the FTO/YTHDF2/TRIB3 axis mediates autophagy which suggests a critical role for the pathogenesis of diabetic wound. Our study provides a potential therapeutic strategy for diabetic wound healing.

## Materials and methods

### Cell culture

The human keratinocyte cell lines HaCaT, NHEK, and 293 T were acquired from the American Type Culture Collection and authenticated through DNA fingerprinting. Human keratinocyte cell lines were cultured in Minimum Essential Medium (MEM) containing 10% fetal bovine serum. In experiments, keratinocytes were exposed to different glucose concentrations (Sigma-Aldrich, G6152) and mannitol (Sigma-Aldrich, M4125). Actinomycin D (Dactinomycin) and FB23-2 were obtained from Selleck (Houston, TX, USA).

### Human tissue samples and ethics statement

We collected diabetic skin tissues from patients undergoing debridement and amputation surgeries and healthy skin tissues from those without diabetes. Harvested tissues were fixed with paraformaldehyde for 24 h, dehydrated, and embedded in paraffin. The study was performed according to the ethical principles of the Declaration of Helsinki II and was approved by the Institutional Review Board of Affiliated Drum Tower Hospital, Medical School of Nanjing University (Ethics Approval Number: 2024-532-01).

### In vivo wound healing model

C57BL/6 mice, type-II diabetic mice (DB/DB), and matched wild-type mice (DB/WT) (male, 6-8 weeks old) were obtained from Model Animal Research Center of Nanjing University. All animal experiments were authorized by the Animal Care and Use Committee of Nanjing Drum Tower Hospital (Ethics Approval Number: DWSY-22094220) and were conducted according to the guidelines of NIH (USA). In the diabetic group, mice were intraperitoneally injected with streptozotocin (50 mg/kg daily, dissolved in sodium citrate buffer, pH 4.0) five days in a row [44, 45]. Blood glucose levels were maintained at a minimum of 16.7 mM for a duration of 4 weeks. The skin wounds were created two days prior (day -2) by using a 10-mm punch to create a circle on the backs of mice, followed by injecting corresponding reagents onto the circle. We then harvested skin tissues from circular wounds made with a 10-mm punch two days later (day 0). After wound modeling, corresponding reagents were injected on the edges of the circular wounds and photos were taken on days 0, 2, 4, 6, 8, 10 and 12. Wound areas were quantified using ImageJ software (National Institutes of Health, Bethesda, MD, USA). The wound healing rate was calculated as previously described (initial area - final area)/initial area × 100% [9].

### Bioinformatics analysis

The gene expression profile dataset GSE80178 was downloaded from the Gene Expression Omnibus (GEO) database. To compare the expression level of m^6^A-regulators between normal and diabetic wound tissues, total RNA was extracted from the scramble and FTO-knockout HaCaT cells. RNA-seq was performed using Illumina Novaseq™6000. For differential gene expression analysis, false discovery rate (FDR) < 0.05 and log 2 (fold change) ≥ 1 were used as thresholds. Data from the GEO database and RNA-seq was analyzed with the R and R package (V4.2, http://www.bioconductor.org). A string database (http://string-db.org) was used to determine protein associations [46]. Protein-protein interactions (PPIs) were conducted by the Metascape website (http://metascape.org) [47].

### RT-qPCR

Total RNA was extracted using the TRIzol reagent. Complementary DNA (cDNA) was synthesized using 500 ng RNA samples with Evo M-MLV Reverse Transcriptase (Accurate Biotechnology, Hunan, China) with gDNAClean. The SYBR® Green kit (Accurate Biotechnology, Hunan, China) was used for RT-qPCR using a Thermo Scientific instrument. Gene expression was analyzed using the 2^-ΔΔCt^ method and normalized to ACTB. The sequences of the PCR primers used were listed in Table 1.

**Table. 1:** Primer sequences.

Gene	Primer	Sequence
ACTB	Forward	GTGGCCGAGGACTTTGATTG
	Reverse	CCTGTAACAACGCATCTCATATT
FTO	Forward	AACACCAGGCTCTTTACGGTC
	Reverse	TGTCCGTTGTAGGATGAACCC
SQSTM1	Forward	GACTACGACTTGTGTAGCGTC
	Reverse	AGTGTCCGTGTTTCACCTTCC
ATG5	Forward	AGAAGCTGTTTCGTCCTGTGG
	Reverse	AGGTGTTTCCAACATTGGCTC
ATG7	Forward	CTGCCAGCTCGCTTAACATTG
	Reverse	CTTGTTGAGGAGTACAGGGTTTT
ULK1	Forward	CCAGAGCAACATGATGGCG
	Reverse	CCTTCCCGTCGTAGTGCTG
TRIB3	Forward	AAGCGGTTGGAGTTGGATGAC
	Reverse	CACGATCTGGAGCAGTAGGTG
YTHDF2	Forward	CCTTAGGTGGAGCCATGATTG
	Reverse	TCTGTGCTACCCAACTTCAGT

### WB

Protein was extracted using lysis buffer (Solarbio, Beijing, China) containing protease inhibitor, phosphatase inhibitor, and PMSF. The mixture of the loading buffer (Beyotime Biotechnology, Shanghai, China) and protein samples was heated to 95 °C for 10 min. We separated proteins by SDS-PAGE gels and transferred them to polyvinylidene fluoride (PVDF) membranes. The membranes were then blocked with 5% nonfat milk. After incubating with primary antibodies (Table 2) at 4 °C overnight, secondary antibodies were incubated at 25 °C for 1 h. Protein bands were measured using enhanced chemiluminescence (ECL, Vazyme, Nanjing, China).

**Table. 2:** Primary antibodies used in this study.

Target	Application	Supplier	Catalog Number
FTO	Western blot/IHC/IF	Abcam	ab126605
FTO	Western blot/IHC/IF	Novus	NBP1-77021
m6A	Dot blot/IF	Synaptic Systems	202 003
cytokeratin	IF	ORIGENE	BP5069
SQSTM1	Western blot/CO-IP/IF	CST	#23214
LC3A/B	Western blot/IHC	CST	#12741
TRIB3	CO-IP	Proteintech Group	13300-1-AP
TRIB3	Western blot	CST	#43043
YTHDF2	Western blot/ CO-IP	Proteintech Group	24744-1-AP
HA-Tag	Western blot	Abmart	M20003
ATG5	Western blot	Abmart	T55766
ATG7	Western blot	Abmart	T55658
ULK1	Western blot	Abmart	T56902
IgG	RIP/Co-IP	Abcam	ab172730
GAPDH	Western blot	Proteintech Group	60004-1-Ig
ACTB	Western blot	Proteintech Group	60008-1-Ig

### TEM

We fixed the cells with 2.5% glutaraldehyde for 5 min. After scraping from the plates, the cell pellets were fixed in 2.5% glutaraldehyde in darkness for 30 min and then post-fixed in 1% osmic acid for 2 h. The transmission electron microscope was used to detect autophagy after dehydrating, embedding, slicing, and staining with 3% uranyl acetate and lead citrate.

### Plasmids and stable cell lines

Cells were transfected with sgRNA targeting negative control (sgNC) (Tsingke, Beijing, China), or an individual gene or the combination of FTO, YTHDF2 using Lipo3000 Transfection Reagent (Invitrogen, Waltham, USA) according to the manufacturer’s instructions. The sequences of sgRNAs are presented in Table 3.

**Table. 3:** sgRNA sequences.

sgRNA	Sequence
sg-FTO-#1	5ʹ-GCCAGAACCTGAGGAGAGAA-3’
	5ʹ-CGGTCTTGGACTCCTCTCTT-3’
sg-FTO-#2	5ʹ-GGCGTGCAGTGAGCGAGGCA-3’
	5ʹ-CCGCACGTCACTCGCTCCGT-3’

### Autophagic flux

Autophagic flux was assessed using mRFP-GFP-LC3 (Genechem, Shanghai, China). HaCaT and NHEK cells were transfected with mRFP-GFP-LC3 lentivirus before related treatment. The fluorescence images were acquired by Leica Thunder Imager.

### RNA dot blot

The RNA samples were denatured at 95 °C for 10 min and cooled on ice for 5 min. After that, the samples were fixed to nylon membranes (Millipore, MA, USA) by ultraviolet (UV) cross-linking. The membrane was blocked with 5% nonfat milk for 1 h and then incubated with anti-m^6^A antibodies (Table 2) overnight at 4 °C. Secondary antibodies were incubated at 25 °C for 1 h. RNA-dots were measured using ECL (Vazyme, Nanjing, China). After being stained with methylene blue, the membranes served as a loading control.

### RIP

RIP assays were conducted using an RIP Kit (Millipore, MA, USA) in accordance with the instruction of the manufacturer. The samples were lysed with lysis buffer containing protease and RNase inhibitor, and then incubated with magnetic beads coated with YTHDF2 antibody (Table 2) overnight at 4 °C.

### MeRIP-qPCR

Total RNA was extracted using RNA Extraction Kit and treated with recombinant DNase I following its corresponding instructions (Accurate Biotechnology, Hunan, China). The MeRIP kit (Millipore, MA, USA) was used according to the instruction of manufacturer. Anti-m^6^A antibodies (or normal IgG as a negative control) were incubated with protein A/G magnetic beads at 25 °C for 4 h under constant rotation. A total of 50 μg RNA was collected as input. The remaining RNA samples were incubated with the magnetic beads-antibody complex at 4 °C overnight. The eluted RNA was purified by RNA Extraction Kit. RT-qPCR was performed as described above.

### Co-IP

After resuspending in IP lysis buffer (Millipore, MA, USA), magnetic beads containing protein A/G were incubated at 25 °C for 2 h with 5 μg TRIB3 or SQSTM1 antibody (or normal IgG as a negative control) (Table 2). The 10% of cell lysate was collected to be used as input. We added the remainder of the lysate to the beads-antibody complex in IP buffer and incubated them overnight at 4 °C. After washing with IP buffer, protein was extracted from beads and heated with loading buffer at 95 °C for 10 min. WB was performed as previously described.

### Hematoxylin-eosin (HE) staining

The fixed tissues were stained with hematoxylin and eosin. Epidermal thickness was determined by ImageJ software analysis throughout each image.

### IHC staining

Skin sections were prepared from paraffin-embedded samples. The slides were baked at 65 °C for 2 h and then rehydrated for 10 min. The slides were submerged in sodium citrate (Solarbio, Beijing, China) solution and heated to 95 °C for antigen retrieval. Slides were blocked with 5% BSA for 1 h at 25 °C. Following incubation with primary antibody (Table 2) overnight, slides were incubated with secondary antibody at 37 °C for 1 h. Images were captured and five random visual fields of each section were selected to conduct histological analysis. The immunoreactive score (IRS score) was calculated by multiplying the percentage of positive cells and immunostaining intensity [48].

### Actinomycin D treatment

Actinomycin D (Selleck, TX, USA) was dissolved in dimethylsulfoxide (DMSO) to 5 mg/ml and diluted with phosphate buffer to maintain a final concentration of 5 μg/ml to inhibit mRNA transcription. The cells were harvested, and the culture medium containing Actinomycin D was replaced one day later. The total RNA was extracted at 0, 3, and 6 h post actinomycin D treatment and used for RT-qPCR.

### Wound-healing assay

Cells were seeded and then scratched with a 200 μl pipette tip after cells attached. Images were obtained at 0 h using microscope. Following cell culture for 20 h, another set of wound images was captured. ImageJ software was used to measure the wound widths, which were then normalized and presented as percentages of the wound measured at 0 h.

### Cell apoptosis

Cells were trypsinized and obtained by centrifugation. Before flow cytometry analysis, cells were resuspended in binding buffer and stained with Annexin V-FITC and propidium iodide (PI) for 15 min in darkness.

### Immunofluorescence

For staining of cells and tissue sections, sections were permeabilized by 0.1% Triton (Beyotime Biotechnology, Shanghai, China) for 20 min. After blocking with 5% normal goat serum, cells and tissues were incubated at 4 °C with primary antibodies (Table 2). Next, sections were washed with 0.1% Triton and incubated with secondary antibodies for 1 h. For RNA-specific m^6^A staining, DNA on the tissue slide was treated by DNase I [49]. In the end, sections were fixed with DAPI (Invitrogen, CA, USA) and visualized under Leica Thunder Imager.

### Luciferase assay

The Luciferase activity was performed using a Dual-Luciferase Assay Kit (Vazyme, Nanjing, China). The 3’UTR of human TRIB3 was amplified by PCR using genomic DNA as a template. We cloned the NCBI reference sequence NM_021158 into the pmirGLO vector using SacI and SalI restriction enzymes. Three putative m^6^A recognition sites were identified in 3’UTR. The firefly luciferase activity values were normalized to the renilla luciferase activity values that reflect expression efficiency.

### Statistical analysis

Statistical analysis was performed in R (version 4.2.2, www.r-project.org), SPSS version 22 (SPSS Inc, IL, USA), and GraphPad Prism v. 8.01 (GraphPad Software, CA, USA). Categorical variables were compared for significance using the χ2 or Fisher’s exact test, and continuous variables were conducted using the Student’s t-test. Spearman correlation analysis was used to analyze the relationship between two variables. Results were reported as means ± SD or SEM obtained from three or more independent experiments. All statistical tests were two-sided and considered significant at *P* < 0.05, *P* < 0.01, *P* < 0.001, and *P* < 0.0001. Figures 2L, 6I and 8 were drawn by Figdraw (www.figdraw.com, ID: RWSUOc44c4, ISUPR074d0 and UUAIT6a943).

## Supplementary information

SFig1, SFig2, SFig3, SFig4

Fig1, Fig2, Fig3, Fig4, Fig5, Fig6, Fig7

## Data Availability

All data used to support the findings of this study are included within the article. The method sections describe the software and resources used for analysis and plotting. The corresponding author can provide any other data upon reasonable request.
