# Supplementary material for: RNA N6-methyladenosine demethylase FTO promotes diabetic wound healing through TRIB3-mediated autophagy in an m6A-YTHDF2-dependent manner
Source: Cell Death Dis. 2025 Mar 29;16(1):222. doi: 10.1038/s41419-025-07494-3 (PMC11954964; doi:10.1038/s41419-025-07494-3)
Supplement: Supplementary file 1 — SFig1, SFig2, SFig3, SFig4 [file 41419_2025_7494_MOESM1_ESM.docx]

SFig.1


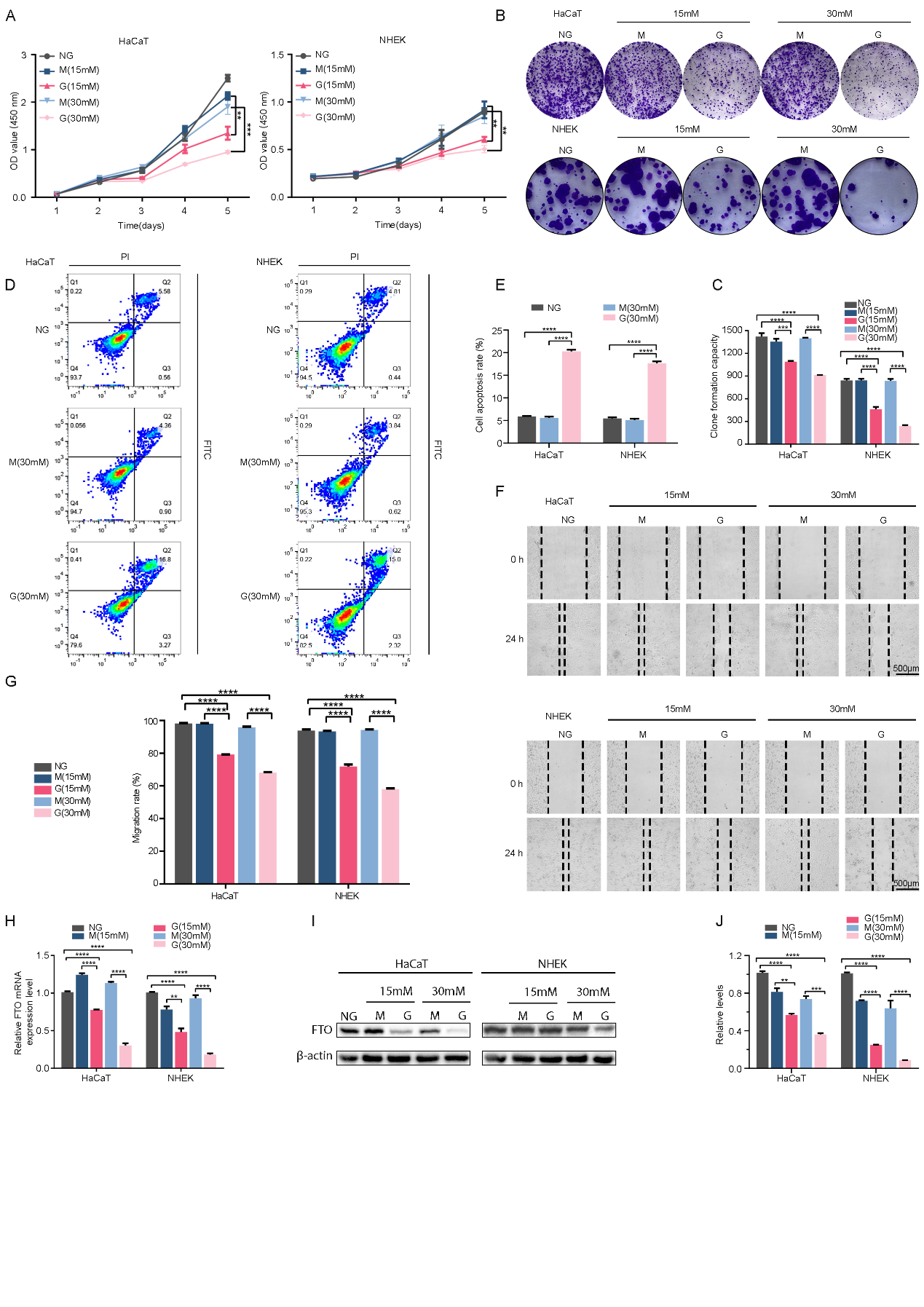


SFig.1 High glucose induced downregulation of FTO and impairment of proliferation and migration in keratinocytes. (A-C) CCK-8 and colony formation assays were applied to evaluate proliferation abilities in HaCaT and NHEK cells treated with NG (normal glucose: 5.6 mM of glucose), 15 mM G (mid-high glucose: 15 mM of glucose), 30 mM G (high glucose: 30 mM of glucose), 15 mM M (15 mM: 5.6 mM of glucose + 9.4 mM of mannitol), and 30 mM M (5.6 mM of glucose + 24.4 mM of mannitol). (D, E) Flow cytometry analysis was used to determine cell apoptosis rates of the HaCaT and NHEK cells treated with NG, 30 mM G, and 30 mM M (mannitol osmotic control) for 72 h. Quantifications of cell apoptosis rates are shown (n = 3). (F, G) Representative images of in vitro wound-healing assays in HaCaT and NHEK cells. The cells were scratched 72 h after treated with NG, 30 mM G, and 30 mM M. Images were captured at 0 h and 24 h after the scratch. Semi-quantitative analysis of wound-healing assays is shown (n = 3). (H) RT-qPCR analysis of FTO mRNA expression levels in HaCaT and NHEK cells treated with NG, 15 mM G, 15 mM M, 30 mM G, and 30 mM M for 72 h (n = 3). ACTB (actin beta) was used as an internal control. (I, J) Western blot analysis of FTO expression levels in HaCaT and NHEK cells treated with NG, 15 mM G, 15 mM M, 30 mM G, and 30 mM M for 72 h. Quantification results of FTO protein expression levels are shown (n = 3). The data are presented as the mean ± SD or mean ± SEM (A, E, C, G, H, J), and *P*-values of all data by a two-tailed unpaired t-test are indicated. **P* < 0.05, ***P* < 0.01, ****P* < 0.001, *****P* < 0.0001.

SFig.2


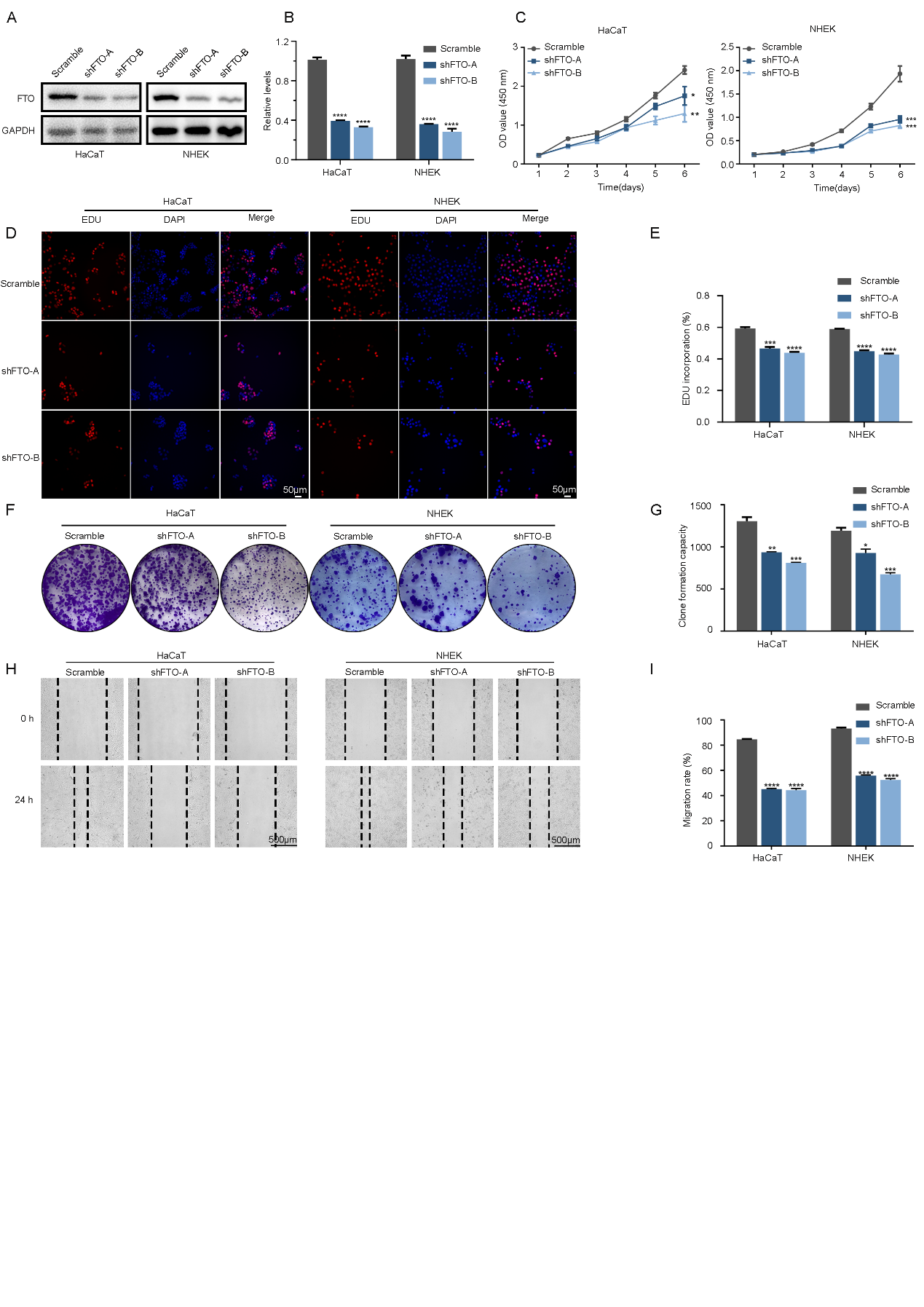


SFig.2 Silencing FTO inhibited the proliferation and migration of keratinocytes. (A, B) Immunoblot analysis to verify the decrease in FTO protein levels in scramble and FTO knockdown HaCaT and NHEK cells. Quantification results of FTO protein expression are shown (n = 3). (C-E) Silencing FTO expression decreased proliferation, as reflected by the CCK-8 and colony formation assay results. Semi-quantitative analysis of colony formation assays is shown (n = 3). (F, G) The EdU incorporation assay was conducted in scramble and FTO knockdown HaCaT and NHEK cells to detect cell proliferation, and the EdU (red) positive cells were semi-quantitated (n = 3; Scale bar, 50 μm). (H, I) HaCaT and NHEK cells were infected with scramble or two different FTO knockdown viruses, and images were captured at 0 h and 24 h after the scratch. Semi-quantitative analysis of wound-healing assays is shown (n = 3; Scale bar, 500 μm). The data are presented as the mean ± SD or mean ± SEM (B, C, E, G, I), and *P*-values of all data by a two-tailed unpaired t-test are indicated. **P* < 0.05, ***P* < 0.01, ****P* < 0.001, *****P* < 0.0001.

SFig.3


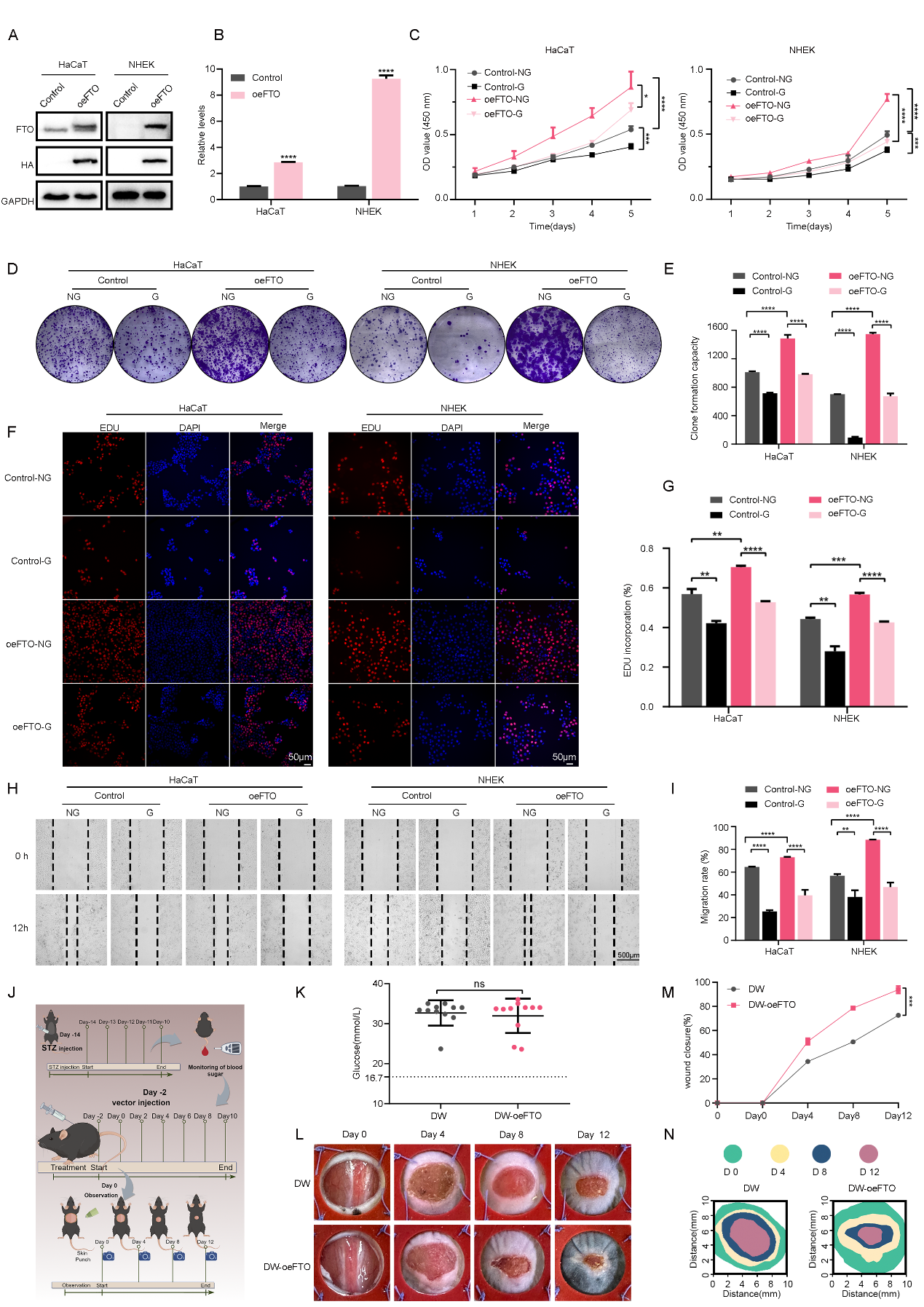


SFig.3 FTO overexpression promoted proliferation and migration of keratinocytes. (A, B) Western blot analysis to verify the transfection efficiency in control and FTO-overexpressing HaCaT and NHEK cells. Quantification results of FTO protein expression are shown (n = 3). (C-E) FTO overexpression in HaCaT and NHEK cells with treatment of normal glucose or high glucose (30 mM) promotes cell proliferation by CCK-8 and colony formation assays. Semi-quantitative analysis of colony formation assays is shown (n = 3). (F, G) Immunofluorescent images of control and FTO-overexpressing HaCaT and NHEK cells with treatment of normal glucose or high glucose (30 mM) for 72 h. EdU (red) was stained for proliferation (n = 3; Scale bars, 50 μm). (H, I) Cell migration capacity of indicated control and FTO-overexpressing HaCaT and NHEK cells with treatment of normal glucose or high glucose (30 mM) for 72 h was measured by scratch wound-healing assays. Images were captured at 0 h and 12 h after the scratch (n = 3; Scale bars, 500 μm). (J) Timeline of in vivo experiments for the STZ, vector injections, and wound healing models of mice. (K) Measurements of blood glucose levels in STZ-treated mice. (L-N) Representative images of cutaneous wounds of diabetic mice injected with control vector and overexpression FTO vetor on days 0, 4, 8, and 12 after wound generation by surgical excision. Rates of wound closure were quantified using ImageJ software and expressed as the percentage of closed wound area (n = 5 per group). The data are presented as the mean ± SD or mean ± SEM (B, C, E, G, I, K, M), and *P*-values of all data by a two-tailed unpaired t-test are indicated. **P* < 0.05, ***P* < 0.01, ****P* < 0.001, *****P* < 0.0001.

SFig.4


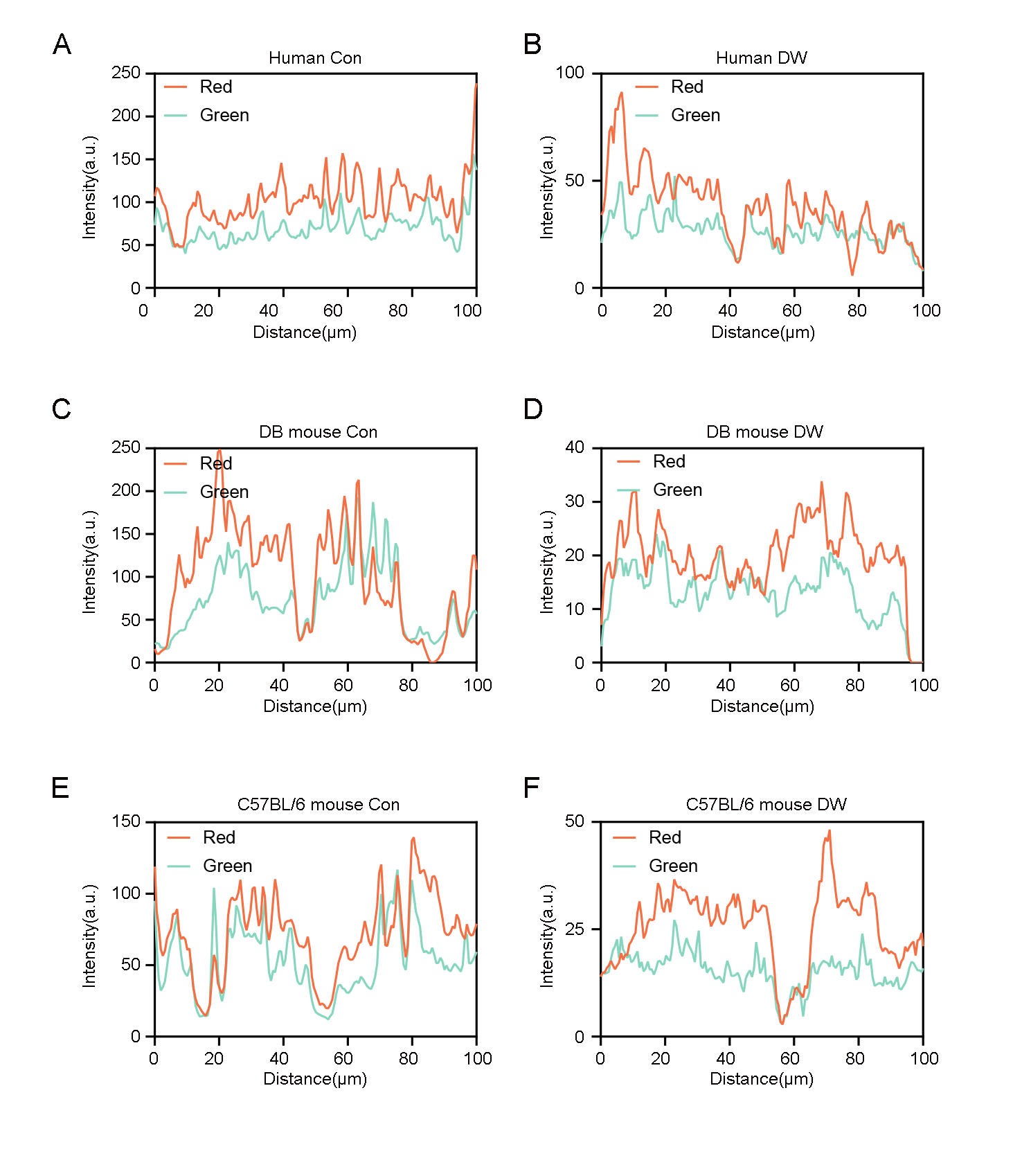


SFig.4

(A-F) Line chart of fluorescence signal positioning analysis in Con and DW tissues (n = 5 per group; Scale bar, 50 μm).
