# Supplementary material for: RNA N6-methyladenosine demethylase FTO promotes diabetic wound healing through TRIB3-mediated autophagy in an m6A-YTHDF2-dependent manner
Source: Cell Death Dis. 2025 Mar 29;16(1):222. doi: 10.1038/s41419-025-07494-3 (PMC11954964; doi:10.1038/s41419-025-07494-3)
Supplement: Supplementary file 2 — Fig1, Fig2, Fig3, Fig4, Fig5, Fig6, Fig7 [file 41419_2025_7494_MOESM2_ESM.pdf]

Fig1

Human

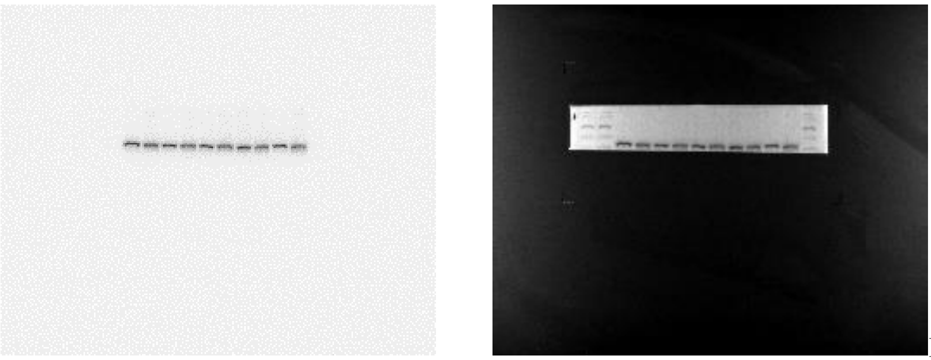

FTO

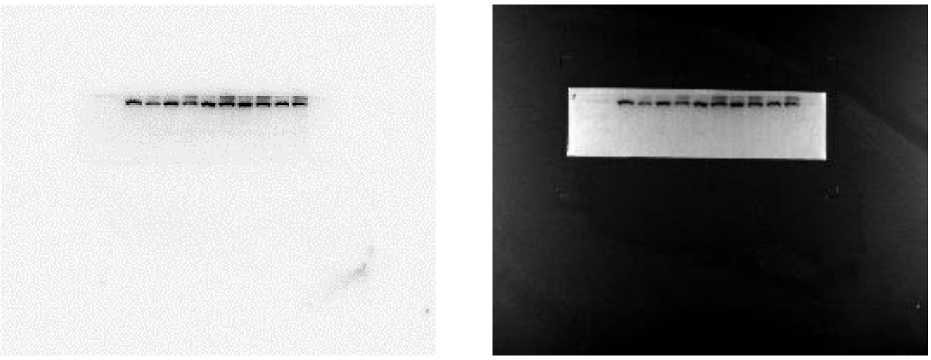

β-actin

DBmouse

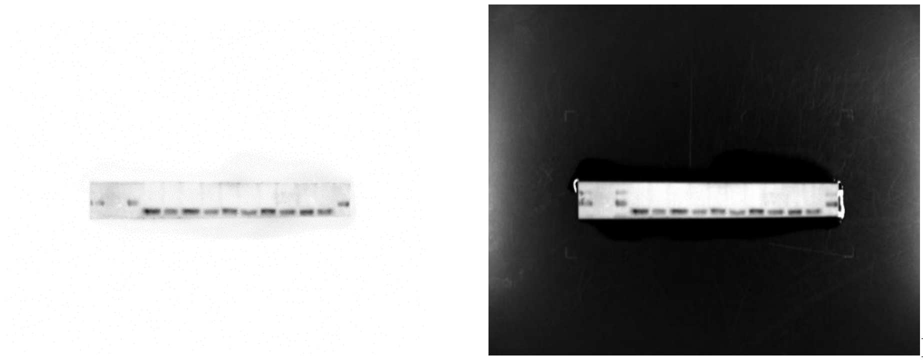

FTO

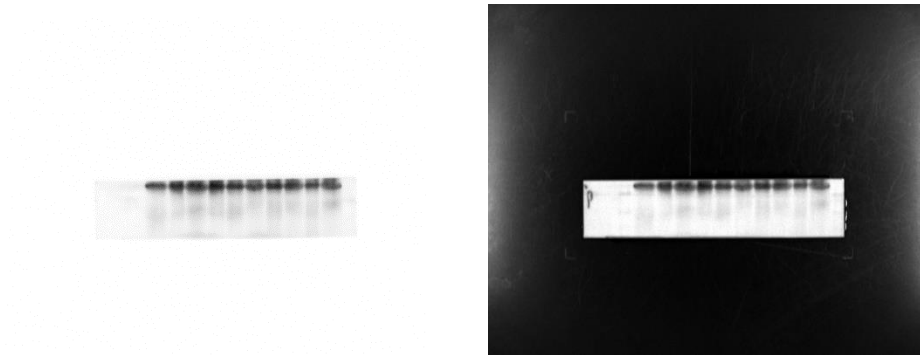

β-actin

Fig2

HaCaT

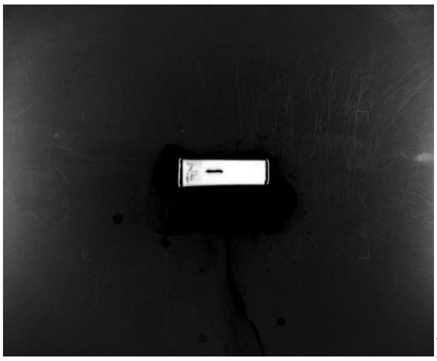

FTO

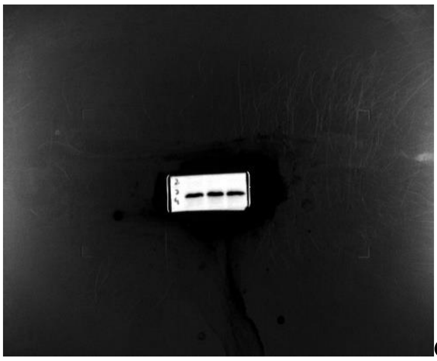

GAPDH

NHEK

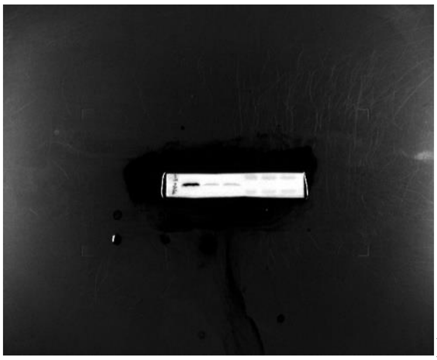

FTO

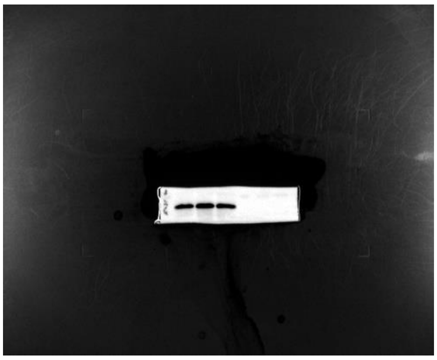

GAPDH

Fig3

HaCaT

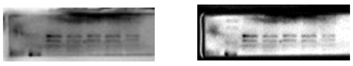

SQSTM1

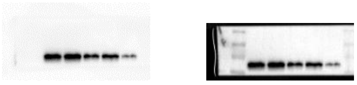

FTO

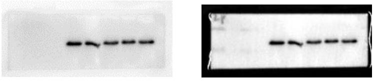

β-actin

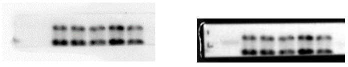

LC3

NHEK

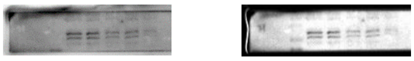

SQSTM1

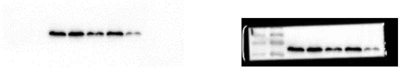

FTO

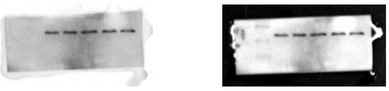

β-actin

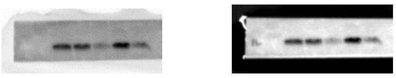

LC3

Fig4

Fig4A

HaCaT

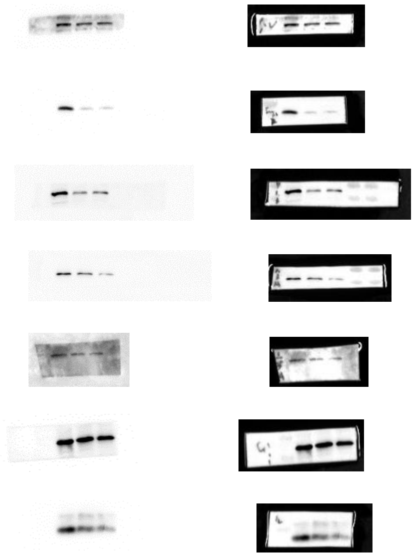

ULK1

FTO

SQSTM1

ATG5

ATG7

GAPDH

LC3

NHEK

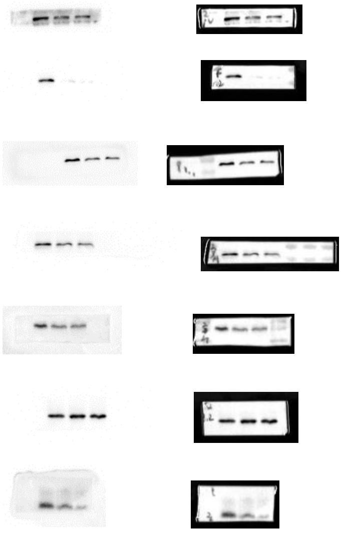

ULK1

FTO

SQSTM1

ATG5

ATG7

GAPDH

LC3

Fig4B

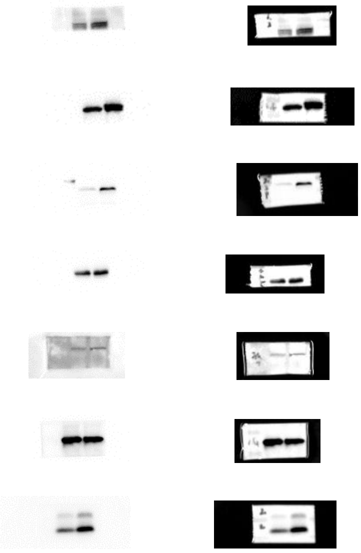

ULK1

FTO

SQSTM1

ATG5

ATG7

GAPDH

LC3

NHEK

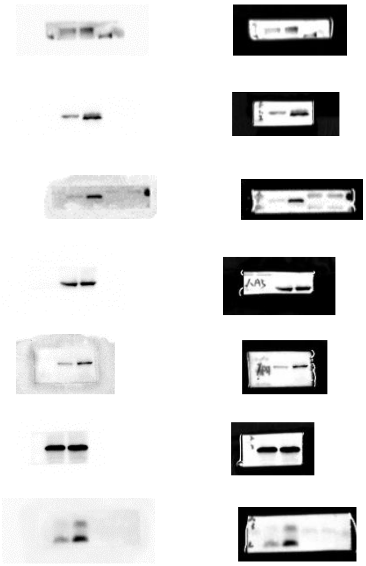

ULK1

FTO

SQSTM1

ATG5

ATG7

GAPDH

LC3

Fig5

HaCaT

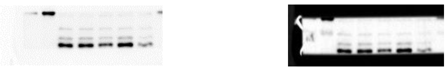

TRIB3

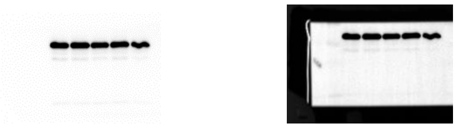

GAPDH

NHEK

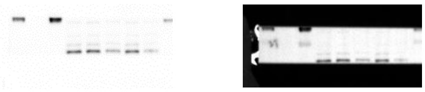

TRIB3

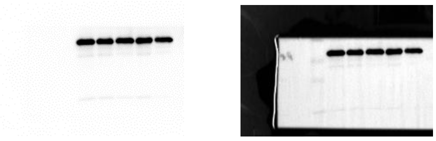

GAPDH

Fig5

HaCaT

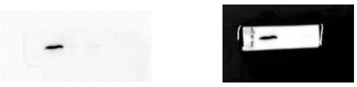

FTO

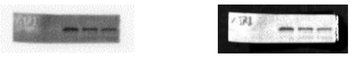

TRIB3

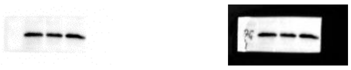

GAPDH

NHEK

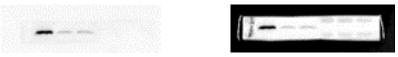

FTO

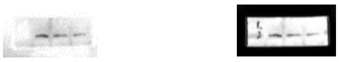

TRIB3

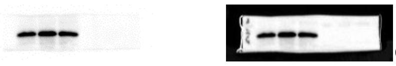

GAPDH

Fig5

HaCaT

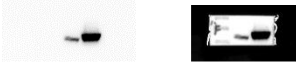

FTO

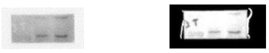

TRIB3

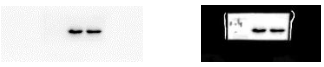

GAPDH

NHEK

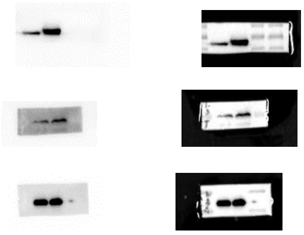

FTO

TRIB3

GAPDH

Fig5

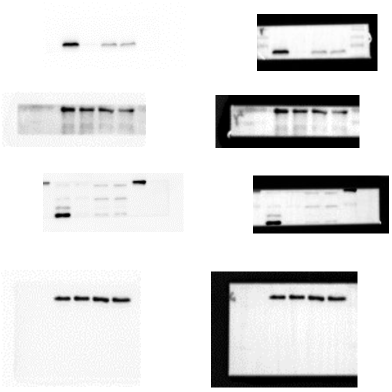

FTO

YTHDF2

TRIB3

GAPDH

Fig7

HaCaT

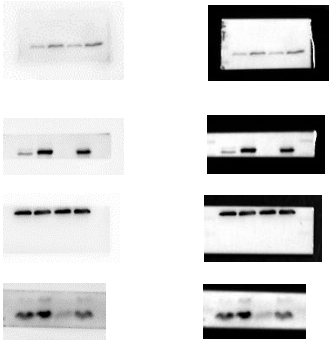

SQSTM1

TRIB3

GAPDH

LC3

NHEK

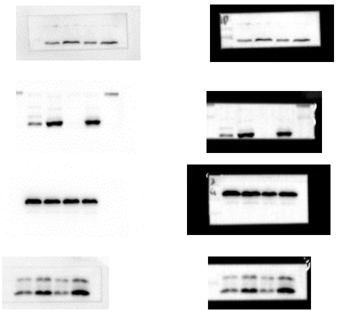

SQSTM1

TRIB3

GAPDH

LC3

Well Positi	Sample Na	Target Na	CT
A1	Sample 1	actb	16.70076
A2	Sample 1	actb	16.66537
A3	Sample 1	actb	16.69848
A4	Sample 2	actb	16.19262
A5	Sample 2	actb	16.15135
A6	Sample 2	actb	15.85655
A7	Sample 3	actb	16.00403
A8	Sample 3	actb	16.28124
A9	Sample 3	actb	16.2976
D1	Sample 1	ATG5	24.90967
D2	Sample 1	ATG5	24.78377
D3	Sample 1	ATG5	24.80936
D4	Sample 2	ATG5	24.71834
D5	Sample 2	ATG5	24.61492
D6	Sample 2	ATG5	24.55365
D7	Sample 3	ATG5	24.71061
D8	Sample 3	ATG5	24.75643
D9	Sample 3	ATG5	24.85178
Well Positi	Sample Na	Target Na	CT
C2	Sample 4	actb	18.74667
C3	Sample 4	actb	18.68116
C4	Sample 4	actb	18.61935
C5	Sample 5	actb	18.06858
C6	Sample 5	actb	18.09495
C7	Sample 5	actb	18.17686
C9	Sample 6	actb	18.2605
C10	Sample 6	actb	18.48522
C11	Sample 6	actb	18.41403
D1	Sample 4	fto	26.75844
D2	Sample 4	fto	26.59148
D3	Sample 4	fto	26.64538
D4	Sample 4	fto	26.69655
D5	Sample 5	fto	27.96025
D7	Sample 5	fto	27.96424
D8	Sample 5	fto	27.98589
D9	Sample 6	fto	28.22644
D11	Sample 6	fto	28.28611
D12	Sample 6	fto	28.2038
Well Positi	Sample Na	Target Na	CT
A1	HSC	ACT	18.85651
A2	HSC	ACT	18.81771
A3	HSC	ACT	18.74224
A4	HSC	ACT	18.84097
A5	HS15M	ACT	20.59719
A6	HS15M	ACT	20.64863
A7	HS15M	ACT	20.66981
A8	HS15M	ACT	20.6662
A9	HS15G	ACT	19.35352
A10	HS15G	ACT	19.12629
A11	HS15G	ACT	19.19913
A12	HS15G	ACT	19.1373
C1	HS30M	ACT	18.44364
C2	HS30M	ACT	18.46556
C3	HS30M	ACT	18.4207
C4	HS30M	ACT	18.48803
C5	HS30G	ACT	17.88003
C6	HS30G	ACT	17.805

C7	HS30G	ACT	18.21348
C8	HS30G	ACT	18.10695
B1	HSC	F	27.37736
B2	HSC	F	27.17053
B3	HSC	F	27.40709
B4	HSC	F	27.41679
D1	HS15M	F	26.42646
D2	HS15M	F	26.55412
D3	HS15M	F	26.19137
D4	HS15M	F	26.41403
D5	HS15G	F	26.27205
D6	HS15G	F	26.72196
D7	HS15G	F	26.94227
D8	HS15G	F	26.51526
B9	HS30M	F	27.43495
B10	HS30M	F	27.70442
B11	HS30M	F	27.85338
B12	HS30M	F	26.72615
B5	HS30G	F	31.69703
B6	HS30G	F	29.86814
B7	HS30G	F	30.92836
B8	HS30G	F	32.85444
E1	HNC	ACT	16.61759
E2	HNC	ACT	16.63484
E3	HNC	ACT	16.5381
E4	HNC	ACT	16.59975
E5	HN15M	ACT	16.73099
E6	HN15M	ACT	16.7914
E7	HN15M	ACT	16.7989
E8	HN15M	ACT	16.76872
E9	HN15G	ACT	16.32929
E10	HN15G	ACT	15.71667
E11	HN15G	ACT	16.03536
E12	HN15G	ACT	15.98906
G1	HN30M	ACT	17.10946
G2	HN30M	ACT	17.18853
G3	HN30M	ACT	17.09505
G4	HN30M	ACT	17.10048
G5	HN30G	ACT	16.74324
G6	HN30G	ACT	16.51304
G7	HN30G	ACT	16.60396
G8	HN30G	ACT	16.64885
F1	HNC	F	24.52289
F2	HNC	F	24.45813
F3	HNC	F	24.57449
F4	HNC	F	24.33791
H1	HN15M	F	25.4404
H2	HN15M	F	25.25788
H3	HN15M	F	25.31342
H4	HN15M	F	25.42434
H5	HN15G	F	25.70129
H6	HN15G	F	25.81454
H7	HN15G	F	25.73585
H8	HN15G	F	25.05871
F5	HN30M	F	24.93064
F6	HN30M	F	24.58767
F7	HN30M	F	24.8061
F8	HN30M	F	24.69134

F9	HN30G	F	26.67369
F10	HN30G	F	26.53223
F11	HN30G	F	26.35756
F12	HN30G	F	25.92057
Well Positi	Sample Na	Target Na	CT
E1	hs	A	16.89605
E2	hs	A	17.06505
E3	hs	A	16.95898
E4	k1	A	17.49069
E5	k1	A	17.46731
E6	k1	A	17.49492
E7	k2	A	17.48492
E8	k2	A	17.44315
E9	k2	A	17.70389
F1	hs	AT7	29.26611
F2	hs	AT7	29.92473
F3	hs	AT7	29.56768
F4	k1	AT7	30.6257
F5	k1	AT7	31.09775
F6	k1	AT7	31.2595
F7	k2	AT7	31.69956
F8	k2	AT7	31.62998
F9	k2	AT7	29.98635
G1	hs	P62	25.37516
G2	hs	P62	25.2879
G3	hs	P62	25.20704
G4	k1	P62	26.76388
G5	k1	P62	26.61381
G6	k1	P62	26.82541
G7	k2	P62	26.67555
G8	k2	P62	26.46221
G9	k2	P62	26.65298
G10	k2	P62	26.6706
H1	hs	ULK	28.20377
H2	hs	ULK	28.05067
H3	hs	ULK	28.16987
H4	k1	ULK	29.16235
H5	k1	ULK	29.51064
H6	k1	ULK	29.51094
H7	k2	ULK	29.84795
H8	k2	ULK	29.8443
H9	k2	ULK	29.79319
Well Positi	Sample Na	Target Na	CT
A1	Sample 1	a	16.55077
A2	Sample 1	a	16.49275
A3	Sample 1	a	16.49046
A4	Sample 1	a	16.43202
A5	Sample 2	a	15.58704
A6	Sample 2	a	15.59464
A7	Sample 2	a	15.60277
A8	Sample 2	a	15.71145
A9	Sample 3	a	15.57503
A10	Sample 3	a	15.76054
A11	Sample 3	a	15.70965
A12	Sample 3	a	15.70743
B1	Sample 1	b	26.59463
B2	Sample 1	b	26.57192
B3	Sample 1	b	26.66246

B4	Sample 1	b	26.48577
B5	Sample 2	b	26.97268
B6	Sample 2	b	27.02488
B7	Sample 2	b	27.13073
B8	Sample 2	b	27.05988
B9	Sample 3	b	26.98851
B10	Sample 3	b	26.91173
B11	Sample 3	b	26.9247
B12	Sample 3	b	26.94164

Well Positi	Sample Na	Target Na	CT
A1	hs	act	16.56353
A2	hs	act	16.41045
A3	hs	act	16.2712
A4	hsha	act	15.83038
A5	hsha	act	15.83983
A6	hsha	act	15.81339
A7	hn	act	16.37577
A8	hn	act	16.35198
A9	hn	act	16.23555
A10	hnha	act	14.78852
A11	hnha	act	14.46755
A12	hnha	act	14.90325
B1	hs	f	26.20636
B2	hs	f	26.06519
B3	hs	f	26.21478
B4	hsha	f	20.64985
B5	hsha	f	20.68309
B6	hsha	f	20.82381
B7	hn	f	25.42634
B8	hn	f	25.3088
B9	hn	f	25.42973
B10	hnha	f	18.12243
B11	hnha	f	18.18663
B12	hnha	f	17.8248
C1	hs	p62	25.13725
C2	hs	p62	25.03646
C3	hs	p62	24.90943
C4	hsha	p62	23.82465
C5	hsha	p62	23.85257
C6	hsha	p62	23.92141
C7	hn	p62	25.44484
C8	hn	p62	25.39478
C9	hn	p62	25.44756
C10	hnha	p62	22.59791
C11	hnha	p62	22.113
C12	hnha	p62	22.62422
D1	hs	lc3b	23.60133
D2	hs	lc3b	23.44683
D3	hs	lc3b	23.15645
D4	hsha	lc3b	22.85028
D5	hsha	lc3b	22.76387
D6	hsha	lc3b	22.93682
D7	hn	lc3b	22.56067
D8	hn	lc3b	21.58268
D9	hn	lc3b	22.00917
D10	hnha	lc3b	22.19989
D11	hnha	lc3b	22.20092
D12	hnha	lc3b	21.94324

E1	hs	ulk1	28.10311
E2	hs	ulk1	27.92526
E3	hs	ulk1	27.65164
E4	hsha	ulk1	25.6094
E5	hsha	ulk1	25.78612
E6	hsha	ulk1	25.65503
E7	hn	ulk1	25.38515
E8	hn	ulk1	25.05172
E9	hn	ulk1	25.14221
E10	hnha	ulk1	23.25536
E11	hnha	ulk1	23.36469
E12	hnha	ulk1	23.26491
F1	hs	bec1	26.42092
F2	hs	bec1	26.0528
F3	hs	bec1	26.02249
F4	hsha	bec1	25.09083
F5	hsha	bec1	24.99444
F6	hsha	bec1	24.92032
F7	hn	bec1	24.64935
F8	hn	bec1	24.7552
F9	hn	bec1	24.51035
F10	hnha	bec1	23.47305
F11	hnha	bec1	23.66158
F12	hnha	bec1	23.41072
G1	hs	a5	25.29947
G2	hs	a5	25.46026
G3	hs	a5	25.261
G4	hsha	a5	23.61788
G5	hsha	a5	23.26442
G6	hsha	a5	23.54134
G7	hn	a5	26.59572
G8	hn	a5	26.01796
G9	hn	a5	26.24998
G10	hnha	a5	23.61804
G11	hnha	a5	23.55831
G12	hnha	a5	23.7361
H1	hs	a7	28.55224
H2	hs	a7	28.7712
H3	hs	a7	28.9615
H4	hsha	a7	27.51411
H5	hsha	a7	27.96892
H6	hsha	a7	27.73206
H7	hn	a7	28.57239
H8	hn	a7	28.66823
H9	hn	a7	28.27205
H10	hnha	a7	25.75779
H11	hnha	a7	26.15897
H12	hnha	a7	26.10563
Well Positi	Sample Na	Target Na	CT
A1	HS	ACT	18.08704
A2	HS	ACT	18.25946
A3	HS	ACT	18.00631
A4	HS	ACT	17.95446
A5	HKO1	ACT	16.7907
A6	HKO1	ACT	17.26226
A7	HKO1	ACT	17.20828
A8	HKO1	ACT	17.1602
A9	HKO2	ACT	16.50692

A10	HKO2	ACT	17.11401
A11	HKO2	ACT	16.77865
A12	HKO2	ACT	17.25961
G1	HS	TIR	26.71152
G2	HS	TIR	26.3141
G3	HS	TIR	26.79148
G4	HS	TIR	27.47574
G5	HKO1	TIR	29.11673
G6	HKO1	TIR	28.93314
G7	HKO1	TIR	29.42108
G8	HKO1	TIR	29.63617
G9	HKO2	TIR	29.02142
G10	HKO2	TIR	29.45325
G11	HKO2	TIR	29.17386
G12	HKO2	TIR	29.38931
Well Positi	Sample Na	Target Na	CT
A1	H	ACT	18.27064
A2	H	ACT	18.2212
A3	H	ACT	18.17946
A4	H	ACT	18.21405
A5	HKO1	ACT	17.31811
A6	HKO1	ACT	17.21412
A7	HKO1	ACT	17.19566
A8	HKO1	ACT	17.24004
A9	HKO2	ACT	17.52375
A10	HKO2	ACT	17.29438
A11	HKO2	ACT	17.25392
A12	HKO2	ACT	17.31155
F1	H	TRI	27.58027
F2	H	TRI	27.47447
F3	H	TRI	27.52592
F4	H	TRI	27.54973
F5	HKO1	TRI	29.63701
F6	HKO1	TRI	29.83163
F7	HKO1	TRI	29.94379
F8	HKO1	TRI	30.10852
F9	HKO2	TRI	29.77491
F10	HKO2	TRI	29.93421
F11	HKO2	TRI	29.88368
F12	HKO2	TRI	29.98279
Well Positi	Sample Na	Target Na	CT
E1	Sample 4	actb	17.98729
E2	Sample 4	actb	17.99471
E3	Sample 4	actb	17.91722
E4	Sample 5	actb	17.56337
E5	Sample 5	actb	17.14114
E6	Sample 5	actb	17.4579
E7	Sample 6	actb	17.42947
E8	Sample 6	actb	17.51655
E9	Sample 6	actb	17.43653
H1	Sample 4	atg5	25.55723
H2	Sample 4	atg5	25.53155
H3	Sample 4	atg5	25.50459
H4	Sample 5	atg5	25.20081
H5	Sample 5	atg5	25.06528
H6	Sample 5	atg5	25.13471
H7	Sample 6	atg5	25.18503
H8	Sample 6	atg5	25.02261

H9	Sample 6	atg5	25.0766
Well Positi	Sample Na	Target Na	CT
B1	WTinput	trib3	28.24389
B2	WTinput	trib3	27.98968
B3	WTinput	trib3	28.15522
B4	WTinput	trib3	28.30262
B5	WTIgG	trib3	37.53381
B6	WTIgG	trib3	33.53329
B7	WTIgG	trib3	33.58
B8	WTIgG	trib3	34.38448
B9	WTip	trib3	28.37958
B10	WTip	trib3	30.87507
B11	WTip	trib3	29.81677
B12	WTip	trib3	30.06224
C1	KOinput	trib3	28.61218
C2	KOinput	trib3	28.57083
C3	KOinput	trib3	28.50035
C4	KOinput	trib3	28.41692
C5	KOIgG	trib3	34.578
C6	KOIgG	trib3	34.94569
C7	KOIgG	trib3	34.83929
C8	KOIgG	trib3	32.45153
C9	KOip	trib3	29.81603
C10	KOip	trib3	32.73491
C11	KOip	trib3	31.64603
C12	KOip	trib3	32.15892
Well Positi	Sample Na	Target Na	CT
A4	WTinput	T1	32.60765
B4	WTinput	T1	31.63053
C4	WTinput	T1	31.60114
D4	WTinput	T1	32.85821
A10	WTip	T1	32.70584
B10	WTip	T1	34.49363
C10	WTip	T1	33.50547
D10	WTip	T1	33.49837
E4	KOinput	T1	30.68193
F4	KOinput	T1	30.06399
G4	KOinput	T1	31.19687
H4	KOinput	T1	30.14009
E10	KOip	T1	31.85466
F10	KOip	T1	30.6379
G10	KOip	T1	31.02174
H10	KOip	T1	31.02791
A5	WTinput	T2	31.35931
B5	WTinput	T2	30.5689
C5	WTinput	T2	30.93546
D5	WTinput	T2	31.80279
A11	WTip	T2	34.00474
B11	WTip	T2	33.90378
C11	WTip	T2	32.50401
D11	WTip	T2	33.03107
E5	KOinput	T2	30.90203
F5	KOinput	T2	30.26604
G5	KOinput	T2	30.63398
H5	KOinput	T2	28.9909
E11	KOip	T2	30.42813
F11	KOip	T2	30.07301
G11	KOip	T2	30.62184

H11	KOip	T2	30.34208
A6	WTinput	T3	30.2188
B6	WTinput	T3	30.17761
C6	WTinput	T3	29.75064
D6	WTinput	T3	30.92511
A12	WTip	T3	30.69502
B12	WTip	T3	31.29562
C12	WTip	T3	31.34875
D12	WTip	T3	30.03722
E6	KOinput	T3	30.25562
F6	KOinput	T3	29.91831
G6	KOinput	T3	30.00062
H6	KOinput	T3	29.52624
E12	KOip	T3	30.40302
F12	KOip	T3	30.37113
G12	KOip	T3	30.46016
H12	KOip	T3	29.92674
